# Prioritisation processes for programme implementation and evaluation in public health: A scoping review

**DOI:** 10.3389/fpubh.2023.1106163

**Published:** 2023-03-27

**Authors:** Shaileen Atwal, Jessica Schmider, Barbara Buchberger, Anelia Boshnakova, Rob Cook, Alicia White, Charbel El Bcheraoui

**Affiliations:** ^1^Economist Impact, Health Policy and Insights, London, United Kingdom; ^2^Evidence-Based Public Health, Centre for International Health Protection, Robert Koch Institute, Berlin, Germany

**Keywords:** health priorities, resource allocation, decision making, decision support techniques, program evaluation (MeSH)

## Abstract

**Background:**

Programme evaluation is an essential and systematic activity for improving public health programmes through useful, feasible, ethical, and accurate methods. Finite budgets require prioritisation of which programmes can be funded, first, for implementation, and second, evaluation. While criteria for programme funding have been discussed in the literature, a similar discussion around criteria for which programmes are to be evaluated is limited. We reviewed the criteria and frameworks used for prioritisation in public health more broadly, and those used in the prioritisation of programmes for evaluation. We also report on stakeholder involvement in prioritisation processes, and evidence on the use and utility of the frameworks or sets of criteria identified. Our review aims to inform discussion around which criteria and domains are best suited for the prioritisation of public health programmes for evaluation.

**Methods:**

We reviewed the peer-reviewed literature through OVID MEDLINE (PubMed) on 11 March 2022. We also searched the grey literature through Google and across key websites including World Health Organization (WHO), US Centers for Disease Control and Prevention (CDC), European Centre for Disease Prevention and Control (ECDC), and the International Association of National Public Health Institutes (IANPHI) (14 March 2022). Articles were limited to those published between 2002 and March 2022, in English, French or German.

**Results:**

We extracted over 300 unique criteria from 40 studies included in the analysis. These criteria were categorised into 16 high-level conceptual domains to allow synthesis of the findings. The domains most frequently considered in the studies were “burden of disease” (33 studies), “social considerations” (30 studies) and “health impacts of the intervention” (28 studies). We only identified one paper which proposed criteria for use in the prioritisation of public health programmes for evaluation. Few prioritisation frameworks had evidence of use outside of the setting in which they were developed, and there was limited assessment of their utility. The existing evidence suggested that prioritisation frameworks can be used successfully in budget allocation, and have been reported to make prioritisation more robust, systematic, transparent, and collaborative.

**Conclusion:**

Our findings reflect the complexity of prioritisation in public health. Development of a framework for the prioritisation of programmes to be evaluated would fill an evidence gap, as would formal assessment of its utility. The process itself should be formal and transparent, with the aim of engaging a diverse group of stakeholders including patient/public representatives.

## 1. Introduction

Programme evaluation is an essential and systematic activity for improving public health actions through useful, feasible, ethical, and accurate methods (1). Finite budgets mean that there is a need to prioritise which programmes are evaluated, however, published discussion of the criteria on which such decisions are made are limited.

National public health institutes and agencies in particular face the need to conduct evidence-based financial resource allocation. Firstly they need to choose programmes to be funded, and subsequently to choose which of the funded programmes are to be evaluated.

Prioritisation aims to balance the allocation or reallocation of resources, particularly in the presence of ongoing challenges such as resource constraints and changing population health needs (2). It is a complex process that is influenced by financial, organisational and political factors, which can sometimes negatively influence decision-making and shift away from ideal priorities (3, 4). Furthermore, the lack of empirical evidence on the prioritisation of public health activities, particularly at a meso/micro level (regional and local public health services) where most public health activities are undertaken, can often lead to suboptimal decision-making and the unnecessary deployment and utilisation of scarce resources (5). With evolving population health needs and the emergence of more effective interventions, there is a greater need to periodically review public health activities to ensure resources are effectively allocated (6).

The complexity of prioritisation is not a new dilemma. The CDC first published its guidelines for setting priorities in public health in 1988 (1). Shortly after this, Vilnius and Dandoy developed an early model which ranked public health issues according to size, urgency, severity of the problem, economic loss, impact on others, effectiveness, propriety, economics, acceptability, legality of solutions, and availability of resources (7). In practise, when public health organisations carry out prioritisation exercises, they may focus on a subset of these criteria. For example, some evidence suggests that the prioritisation criteria used in low- and middle-income countries (LMICs) tend to focus on cost and health benefits, encompassing elements of cost-effectiveness and effectiveness of interventions (8). Disease epidemiology is another common consideration in deciding on public health priorities. For example, decision-makers can consider the burden of disease or other epidemiological indicators collected in surveillance systems when deciding on which disease areas to target. However, the use of solely quantitative criteria is unsustainable and unpragmatic as it does not consider qualitative issues such as the feasibility, reach and ethics of interventions, and therefore it can hinder fair decision-making and allocation of resources (9, 10). Instead a combination of quantitative and qualitative criteria are required (11). For example, use of qualitative approaches such as focus groups and other methods of stakeholder involvement enables the incorporation of broader feasibility and social issues such as public perception of the need for an intervention into the decision-making process (11). This aids consensus building around priorities and should increase ease with which interventions can be implemented, as well as improving social acceptability and uptake.

In this scoping review, we aimed to systematically identify and map the criteria that have been used or suggested for use for prioritisation in public health and discuss criteria that have been or could be used in the prioritisation of public health programmes to be evaluated.

## 2. Methods

### 2.1. Study design

The research questions addressed by this scoping review are:

1. What criteria have been proposed for prioritisation in public health?
2. Which identified criteria have been suggested to be used specifically to prioritise programmes for evaluations?
3. What is the evidence on use and utility of said criteria?

The first question was the focus of the review and was the basis for the development of our literature searches.

This review follows the PRISMA 2020 reporting guidelines and checklist for systematic reviews (12). Further description of the study, its aims and processes are provided in the review protocol (see Supplementary material).

### 2.2. Identification of studies

Our initial scoping searches found that limited additional references were identified through searching both OVID MEDLINE (PubMed) and Embase, so the final database search only encompassed OVID MEDLINE (PubMed) (2002 to 11 March 2022), and we focused on supplemental techniques to identify additional references. Search strategies are provided in the Supplementary material. We utilised supplemental techniques including keyword searches of Google and Google Scholar as well as key websites including World Health Organization (WHO), US Centers for Disease Control and Prevention (CDC), European Centre for Disease Prevention and Control (ECDC), and the International Association of National Public Health Institutes (IANPHI). These searches were conducted in February and March 2022. The terms utilised included “prioritisation” and “public health.” The first 10 pages of results were screened for the Google platforms and the first five pages for other platforms.

We performed reference harvesting and citation tracking for the included papers which considered prioritisation broadly within public health (what we referred to as “generic” prioritisation papers) rather than those which limited their prioritisation to a specific field within public health (for example a single disease area or a disease subgroup such as infectious disease—what we referred to as “specific” prioritisation papers). The focus on papers with a broader scope reflects the broad focus of our research question and the intention that criteria identified could be used for prioritising evaluations across any public health programme. We did not apply geographical or language limits to the search, but only considered studies with full texts available in English, French, or German for inclusion. Searches covered the past 20 years (2002 to 11 March 2022) to encompass the most up-to-date approaches to prioritisation. References were managed in Endnote v.20.

### 2.3. Selection process

We sifted the search results in three stages. An information specialist carried out a first pass sift at title and abstract level to remove non-relevant material and duplicates. Two reviewers carried out a second pass sift at title and abstract level to assess relevance to the review scope, erring on the side of caution for potentially relevant papers. These reviewers also carried out a third pass sift at full text to decide on final inclusions and exclusions. A third, senior reviewer independently assessed all full texts reaching this third pass sift. The review team discussed and reached agreement on any queries that arose during sifting. Reviewers drafted inclusion/exclusion criteria *a priori* based on the review scope with key sifting decisions and clarifications to the scope and inclusion criteria recorded. The inclusion/exclusion criteria are detailed in Table 1. Due to the nature of the review question, we did not exclude studies based on study design, with both empirical studies and non-empirical reports of prioritisation criteria being used or proposed for use in public health eligible for inclusion.

**Table 1:** Inclusion and exclusion criteria.

*Inclusion criteria* Papers reporting single or multiple criteria for prioritisation in public health Papers from any countries Any study designs (including non-empirical descriptions) Published or grey literature Papers published in English, French or German
*Exclusion criteria* Papers published prior to 2002 Papers on prioritisation in healthcare more broadly without specific reference to of government funded (or donor-funded in the case of LMICs) public health Papers describing how to conduct an evaluation Papers that do not make reference to prioritisation of investments/resources Papers on geographical prioritisation, e.g., of regions for use of interventions such as insecticide treated bed nets Prioritisation of approaches (e.g., a global approach versus a national approach to a problem) rather than disease areas or programmes Prioritisation of research questions/areas/topics Papers focusing on methodological approaches for assessing individual criteria or incorporating these into models Prioritisation within very narrow specialised areas of public health, (e.g., water pollutants, specific pathogen subgroups such as zoonoses), biocides, bioweapons, pharmaceuticals Prioritisation of population groups or patients for specific interventions (e.g., covid vaccines) or resources (e.g., clinical medical resources in a triage situation) Assessment (e.g., survey) of people’s priorities (e.g., public health professionals) without explicit exploration of specific criteria for priority setting Duplicate publications of the same study

### 2.4. Data extraction

Two reviewers conducted data extraction and cross checked each other’s extractions. These reviewers categorised the prioritisation criteria into conceptual domains and a third reviewer independently repeated this process. Reviewers discussed and reached agreement on any discrepancies in extraction or categorisation. Full data extraction tables are provided in the Supplementary material.

### 2.5. Data categorisation

We extracted data from included studies into an agreed data extraction table. Studies were categorised according to:

- Whether they were empirical (research-based) studies.
- What level prioritisation occurred at (macro – national or international level, meso – regional level, micro – local public health body level).
- Whether prioritisation was carried out across diseases/areas in public health (generic) or within a smaller disease or programme area (specific).
- Whether the units being prioritised were diseases/risk factors or programmes/interventions.
- Which criteria for prioritisation of interventions for implementation or evaluation were used.
- Whether the prioritisation approach being described was being practically applied in a real prioritisation exercise in a public health setting in the study (categorised as an “actual” use), or whether the tool or approach was being described conceptually without description of it being applied in actual priority setting (categorised as a “theoretical” description).

### 2.6. Quality assessment

Included empirical studies utilised a variety of study designs, both quantitative and qualitative. As such, we selected a tool which had been designed for use in systematic reviews of health services research topics including studies of different designs—the Quality Assessment with Diverse Studies (QuADS) tool (13). A similar systematic review of prioritisation in public health in LMICs had used an earlier version of this tool—the Quality Assessment Tool for Studies with Diverse Designs (QATSDD) (8). The QuADS tool was piloted with studies of each of the included study types, and the approach to assessing each of the 13 questions in the tool recorded to ensure consistency (13). The QuADS tool typically involves scoring studies from 0 to 3 (lowest to highest quality) on each question. However, quantitative scales may oversimplify the complex issue of quality by implying equivalent weight of the individual questions. Therefore we chose to use a qualitative colour grading system from red (lowest quality) to green (highest quality) rather than quantitative scores, similar to the approach used in the Cochrane risk of bias tool (14).

### 2.7. Data synthesis

We extracted prioritisation criteria as reported in the study, along with additional detail to clarify their meaning and avoid making assumptions in interpreting the authors’ intent. We grouped conceptually related criteria into higher level conceptual domains to facilitate synthesis of the information. We developed a group of higher level domains based on the domains utilised in a systematic review of prioritisation in LMICs (8), with refinements to avoid any gaps, overlap between domains, or unclear/inconsistent groupings. Our final 16 domains are listed with their definitions in Table 2. Additional detail on the criteria included in each provided in the Supplementary material.

**Table 2:** Domains and their definitions.

Domain	Domain definition
Burden of disease	Relates to number of people affected or severity of disease, including measures of health service need/demand/use that are not explicitly translated into health system costs (e.g., risk of admission to hospital). Does not include the economic burden of a disease, which is captured in other domains.
Equity/Fairness/Ethics/Equality	Relates to any issues linked to equity, fairness, ethics or equality, pertaining to the disease/risk factor itself (e.g., whether they disproportionately impact vulnerable groups), or the intervention (e.g., whether it specifically targets and reduces inequity). For example, these issues could relate to characteristics such as gender, sexual orientation, ethnicity, education or wealth, or to reducing inequalities.
Cost-effectiveness/value for money	Relates to explicit consideration or calculation of the balance between costs and benefit of an intervention/programme, e.g., cost effectiveness ratios (ICERs), cost per quality-adjusted life year (QALY), or cost per identified benefit. This includes criteria reported in studies simply as “value for money” or “cost-effectiveness” with no further detail.
Budget impact of the intervention/programme	Relates to the budget impact of investment or disinvestment in the programme for the body funding it.
Cost of disease from a health system perspective	Relates to the health system cost of providing care for people with a disease, i.e., economic burden of disease for the health system.
Cost of disease from a patient perspective	Relates to the cost to patients of obtaining care for a disease.
Cost of disease from a societal perspective	Relates to the cost of a disease to wider society, e.g., through Absenteeism (from work or school) Loss of productivity Need for informal care Impact on wider industries and the economy.
Health and wellbeing impacts of intervention/programme	Relates to positive and negative health and wellbeing outcomes of the intervention/programme. For example, effectiveness/efficacy of intervention/programme, including comparative (dis)advantage versus other options, side effects or harms associated with the intervention/programme. Also includes changes in, e.g., knowledge and behaviour, as these have the potential to lead to changes in health and wellbeing outcomes.
Social considerations	Relates to the social aspects of the disease or the intervention/programme. For example, fear/risk perception/stigma of the disease itself, societal acceptability of a programme/intervention, impact of a programme/intervention on wider societal outcomes including community capacity.
Organisational/provider/industry considerations	Relates to the acceptability and impact of the intervention/programme to the public health organisation carrying out the prioritisation, providers such as healthcare professionals or wider healthcare industry bodies. Includes whether the intervention is included in clinical guidelines & practises, or if there is evidence of variation in practise, and whether a disease is difficult to manage. It also includes alignment with organisational priorities/strategy.
Legal & regulatory framework	Relates to any legislative or regulatory requirements or issues relating to the intervention/programme which affect provision/implementation. For example, this includes national level decisions about inclusion of an intervention in provision (e.g., inclusion of a vaccine in the national vaccine programme) which need to be complied with at regional or local level.
Political considerations	Relates to any political considerations, such as alignment with government policy.
International support/donor acceptance	Relates to alignment with international policy or donor strategy/priorities, including acceptability of prioritisation of the disease or of the intervention/programme.
Feasibility of implementation	Relates to the feasibility of implementing an intervention/programme and any factors affecting this. For example, whether an effective prevention or treatment is available for a disease, what proportion of the affected population a programme/intervention could target, the ability to provide quality care, or any capacity constraints or technical issues with providing an intervention/programme. (Does not include any feasibility considerations falling under other domains, e.g., budget impact or legal & regulatory framework).
Current provision of services	Relates to existing provision of services in the community. For example, whether an intervention/programme or alternative measures are being provided in the community, or what access is like to the services.
Evidence considerations	Relating to issues around the evidence-base, such as availability/strength/quality of evidence about the impact of an intervention/programme.

A senior reviewer checked all criteria extractions and domain categorisation of criteria, and any disagreements were resolved through discussion among the team. We summarised the frequency of use of the individual domains using graphical representation, and used narrative descriptions to compare and describe relationships between the domains used and other study characteristics. We discuss the potential impact of reporting bias narratively in the discussion.

## 3. Results

### 3.1. Study selection and characteristics

**Figure 1:**
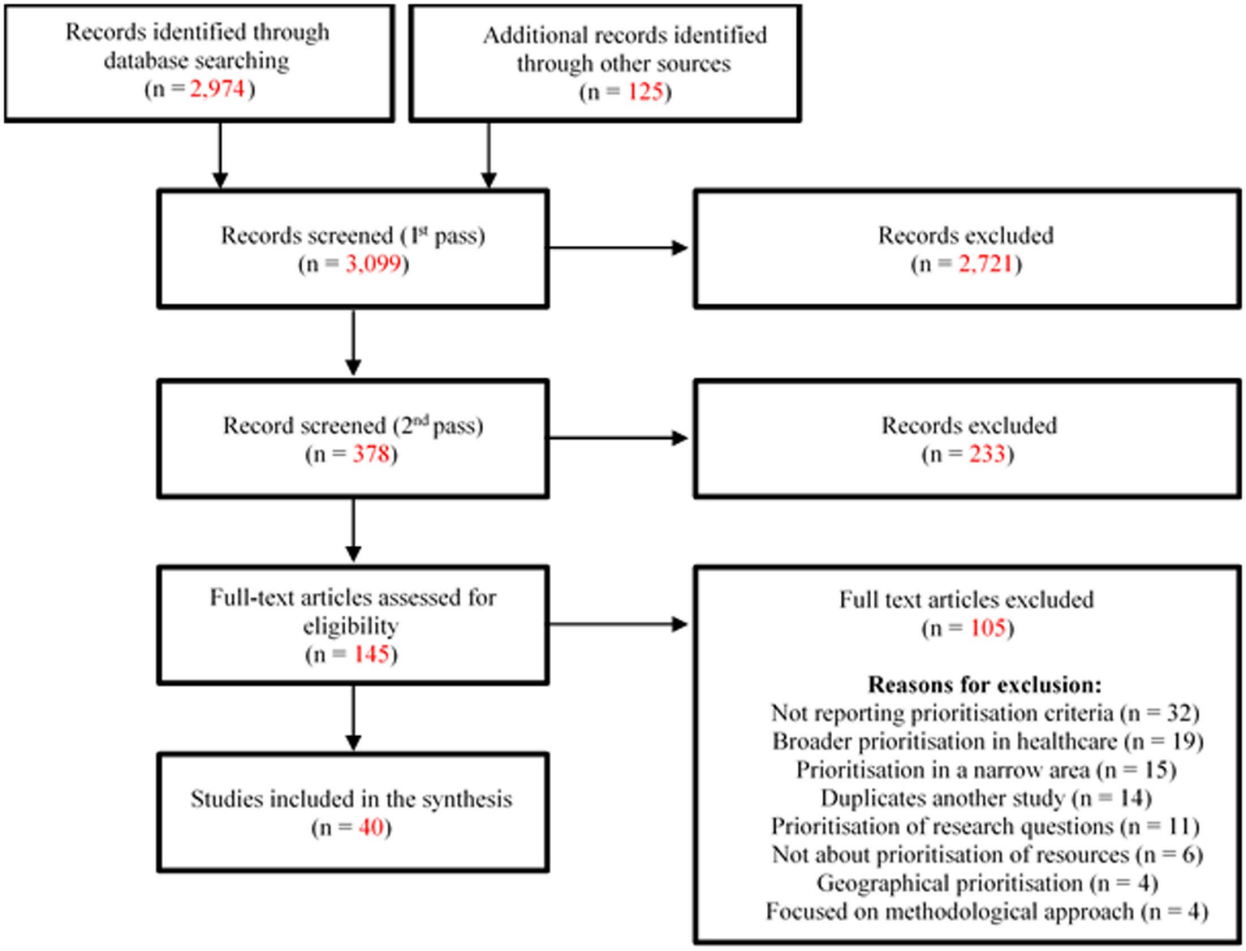
PRISMA flow diagram summarising the search and selection process in this scoping review.

We identified a total of 3,099 unique records through database searches and other supplemental searches. Of these studies, 40 met inclusion criteria and are described further here (3–5, 8, 10, 15–49). A PRISMA flowchart of the literature search is shown in Figure 1. Table 3 summarises key characteristics of the included studies.

**Table 3:** Characteristics of included studies.

*Overall (n = 40 studies)* Empirical (*n* = 30) *Systematic review (n = 4)* *Mixed methods (n = 9)* *Qualitative studies (n = 3)* *Prioritisation exercises (n = 7)* *Model/framework development and case studies/evaluations (n = 7)* Non-empirical (*n* = 10) *Description of proposed prioritisation framework/tool (n = 5)* *Prioritisation tool developed by multinational/national bodies (n = 3)* *Narrative descriptions of a prioritisation process (n = 2)*
*Level at which prioritisation carried out* Macro (*n* = 24) Meso (*n* = 8) Micro (*n* = 16) Not specified (*n* = 2)
*Generic vs. specific* Generic (*n* = 26) Specific (*n* = 14) *Infectious disease interventions (n = 5)* *Pathogens (n = 5)* *Indigenous healthcare programs (n = 1)* *Non-communicable disease control (n = 1)* *Surgical interventions (n = 1)* *Reproductive, maternal and child health interventions (n = 1)*
*Publishing organisations* International/multinational organisations (*n* = 3) National/state health agencies (*n* = 14) Local health authorities (*n* = 7) Donor organisation (*n* = 1) Charity (*n* = 1) Academic institutions (*n* = 11)
*Countries included* Single country perspective (*n* = 27) *USA (n = 9)* *UK (n = 8)* *Germany (n = 3)* *Canada (n = 2)* *Belgium (n = 1)* *Indonesia (n = 1)* *Australia (n = 1)* *Sweden (n = 1)* *Kenya (n = 1)* Global perspective (*n* = 2) Regional or developmental perspective (*n* = 9) *LMICs or resource poor settings (n = 4)* *LMICs in Asia Pacific (n = 1)* *Africa (n = 1)* *EU (n = 1)* *Americas (n = 1)* *OECD (n = 1)*

### 3.2. Quality appraisal

Details of the quality appraisal and its results are provided in Supplementary material. Overall, we found that most empirical studies were judged to be of at least moderate quality. Most studies used appropriate study designs and methods to answer their research aims; these aims and the research setting tended to be well described. However, few studies clearly described recruitment approaches (whether this be, e.g., study flow and inclusion information for reviews, or recruitment of participants for interview) or gave good critical discussions of their strengths and weaknesses.

There is no tool for quality appraisal of non-empirical studies, however, it is worth considering the quality of their content. Some of these pieces were narrative pieces/editorials, and as such these should be considered the authors’ opinions and not a systematic assessment of what criteria are available (42, 44–49). Some of these studies, such as Wani et al. (2020), had very limited reporting on the decision making criteria and processes involved in prioritisation (48). Other non-empirical papers described prioritisation tools, some developed by national or supra-national public health bodies (40, 41, 43). In these papers, the tools were not accompanied by a detailed description of their development, however, this does not mean that the tool or processes used were not robust.

### 3.3. Criteria used or proposed for prioritisation in public health

#### 3.3.1. Frequency of domains considered in prioritisation

We identified over 300 different criteria as being used in public health prioritisation processes. A summary of the mapping of criteria to domains is provided in the Supplementary material.

**Figure 2:**
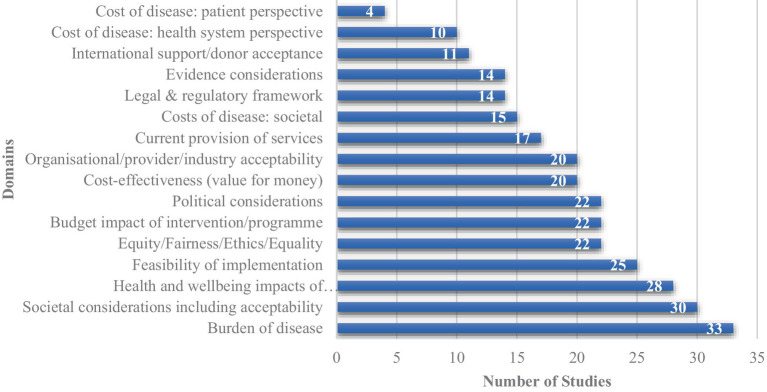
Frequency of prioritisation domains cited in included studies.

Figure 2 shows the frequency of the 16 domains featuring criteria that were elicited from the 40 included studies. Across the included studies, the most reported domains considered were the “burden of disease” (*n* = 33), “social considerations” (*n* = 30), “health impacts of the intervention” (*n* = 28), and “feasibility of implementation” (*n* = 25). While domains encompassing elements of cost such as “cost of disease from a patient perspective” (*n* = 4) were among the least cited, however the combination of all cost domains including “cost-effectiveness,” “budget impact” and cost of disease from a health system, patient & societal perspective were cited across 34 of the 40 studies included in this review (3–5, 8, 10, 15–28, 30–33, 36, 38–43, 46–49).

We found that most of the included studies reported using multiple criteria domains in their prioritisation decisions except for two studies, both of which used modelling focused on a single outcome (33, 34). Only one study described criteria related to all 16 domains, as it was a review which aimed to give a comprehensive list of possible prioritisation criteria (10).

#### 3.3.2. Criteria used in specifically focused versus generic prioritisation

Out of the 40 included studies, we deemed 14 as “specific” as they focused on a particular disease area, type of intervention or population subgroup (4, 16, 18, 19, 25, 27, 28, 31–33, 39, 40, 46, 47). Of these studies, five focused on a particular disease area, in most cases this was an infectious disease (25, 27, 31, 32, 40) and all reported criteria related to the “burden of disease” and “societal costs of disease” among others.

The remaining nine “specific” papers had a greater focus on the prioritisation of specific public health interventions and policies and frequently cited criteria relating to “social considerations” (*n* = 7), as well as “equity/fairness/ethics/equality,” “political considerations” and the “feasibility” and “cost-effectiveness” of the programmes/interventions/policies (*n* = 6 each) (4, 16, 18, 19, 28, 33, 39, 46, 47). Studies categorised as “generic” (*n* = 26) had a broader focus on public health interventions or multiple disease areas/risk factors (3, 5, 8, 10, 15, 17, 20–24, 26, 29, 30, 34–38, 41–45, 48, 49). These studies frequently cited criteria relating to “burden of disease” (*n* = 23), “health impacts of the intervention” (*n* = 22), and “social considerations” (*n* = 19).

#### 3.3.3. Prioritisation criteria for diseases/risk factors compared with programmes/interventions

The most cited criteria in the eight studies that prioritised diseases and/or risk factors pertained to “burden of disease” (*n* = 8), “social considerations” and “societal costs of disease” (*n* = 6 each), whereas “cost-effectiveness” and “cost of disease from a patient perspective” were not considered in any study prioritising diseases (25, 27, 31, 32, 34, 37, 40, 42).

This varied from the 24 studies that prioritised programmes/interventions or policies, which most frequently reported utilising criteria pertaining to the “health impacts of the programme/intervention” domain (*n* = 20) (8, 15, 16, 18–20, 23, 24, 26, 28, 29, 33, 35, 36, 38, 39, 41, 43–49).

#### 3.3.4. Prioritisation criteria for diseases compared with programmes in “generic” versus “specific” studies

When looking at the five studies focusing on prioritisation of diseases, we found that “burden of disease” and “cost of disease from a societal perspective” (mainly work/school absenteeism and productivity loss) were the most cited domains. All these studies focused on prioritisation of infectious disease/pathogens at a macro level (25, 27, 31, 32, 40).

Amongst the three “generic” studies focusing on the prioritisation of diseases or risk factors, “burden of disease” and “social considerations” were the most commonly cited domains (*n* = 2 each) (34, 37, 42).

“Health & well being impacts of the intervention” (*n* = 13) and “burden of disease” (*n* = 12) were the most frequently cited domains amongst the 16 “generic” studies that prioritised programmes/interventions (8, 15, 20, 23, 24, 26, 29, 35, 36, 38, 41, 43–45, 48, 49). In the nine studies that focused on interventions/programmes or policies within specific sub-areas “cost-effectiveness,” “social aspects” (such as public acceptability), and “feasibility of implementation” were the most commonly cited domains (*n* = 6 each) (4, 16, 18, 19, 28, 33, 39, 46, 47). This likely reflects that disease burden is not as useful a criterion in cases where the interventions being prioritised all focus on the same disease or disease area.

#### 3.3.5. Criteria described in theoretical frameworks versus actual prioritisation exercises

Of the 40 included papers, we found that 13 papers reported on either the initial development of a prioritisation framework or tools or discussed proposed criteria for prioritisation; we categorised these as being “theoretical” as they did not describe their use in actual prioritisation exercises (15, 27, 28, 34, 39, 42–49). The high-level grouping domains cited in these papers were “health impacts” (*n* = 10) and “burden of disease” (*n* = 9), “social considerations” (*n* = 9), “equity/fairness/ethics/equality” and “budget impacts” (*n* = 7).

Twelve papers reported testing recommended methods in actual prioritisation exercises (23, 29–33, 35–38, 40, 41). The most common domains considered in these papers were similar to the theoretical frameworks: “burden of disease” (*n* = 10), “health impacts” and “social aspects” (*n* = 7), and “equity/fairness/ethics/equality” (*n* = 6). In addition, “feasibility of implementation” (*n* = 7) was also a commonly used criterion in these papers. “Budget impact” (*n* = 1) was less commonly used in these studies than in the theoretical frameworks. This was in part because several of these papers (*n* = 4) prioritised diseases and not interventions/programmes (as “budget impact” pertained to programmes/interventions specifically), while others looked at “cost-effectiveness (value for money)” rather than budget impact (*n* = 5).

Ten papers used qualitative or observational methods to identify the criteria decision-makers had used in actual public health priority-setting (3–5, 18–22, 24, 26). These papers considered domains such as “burden of disease” (*n* = 10), “budget impact,” “social considerations” and “political considerations” (*n* = 9), followed by “organisational/provider/ industry considerations” (*n* = 8) and “feasibility of implementation” (*n* = 7).

#### 3.3.6. Actors involved in prioritisation and relationship to criteria

We found that decision-makers involved in the prioritisation process varied according to the level at which prioritisation was carried out. For instance, decisions made at micro and meso levels often involved stakeholders from local public health bodies and regional level commissioners (3, 5, 8, 15, 19–24, 26, 29, 30, 37, 41, 43). Macro-level prioritisation was centred on decision-making at a country level and involved national government officials and directors of national public health organisations (4, 8, 15, 17, 18, 25, 28, 31, 32, 35, 36, 38, 40, 47).

Epidemiologists and infectious disease experts were always consulted on the prioritisation of infectious diseases which all occurred at a macro level (25, 27, 31, 32, 40). These studies used similar methods and all cited “burden of disease” and “societal costs of the diseases” (*n* = 5 each) as criteria, followed by “feasibility” (in terms of the preventability and treatability of the disease) and “international support” (*n* = 4 each). Other common criteria were “cost of disease from a health system perspective” and “political considerations” (*n* = 3).

Only three non-review studies explicitly stated that their prioritisation processes involved (or were suggested to involve) some local community/public/patient representatives, and these papers also all cited criteria which fell into the “social considerations” domain such as the impact on service users, caregivers and families, and the local community (20, 29, 43). Some papers reported some, but not consistent, public involvement in priority setting by decision-makers, (8, 20) or suggested that their process could be improved by inclusion of patient/public engagement in the prioritisation process to expand societal perspectives (31). Many other papers reported using criteria which fell into the “social considerations” domain but did not specify whether or how they sought input on these from the community.

Additionally, actors involved in studies which assessed what decision-makers do in actual prioritisation exercises were often officials from local and regional-level (e.g., US state) public health organisations, various health sectors and epidemiologists/public health specialists. These studies most often considered criteria related to “burden of disease” (*n* = 8) as well as “health impacts of the intervention,” “social considerations” and “organisation/provider/healthcare industry considerations” (*n* = 7 each).

#### 3.3.7. Processes used in prioritisation

Overall, 27 of the included studies in this review reported on the approaches or processes used to carry out the prioritisation process such as multi-criteria decision analysis (MCDA), programme budgeting and marginal analysis (PBMA), accountability for reasonableness (A4R) and the Delphi process. Eight of the primary studies as well as in primary studies included in four reviews used MCDA, which was the most used method for prioritisation (8, 10, 15–17, 23, 27, 28, 40, 41, 43, 50). Four studies used the PBMA approach (16, 21, 29, 30). All papers that applied the PBMA or MCDA approach also included multiple stakeholders in the process, sometimes including representatives from civil society, as this is a standard part of these approaches.

Some papers described a less structured approach to prioritisation, and studies which obtained decision-makers’ experiences of prioritisation rarely reported on whether specific processes or approaches were used (3–5, 18–20, 22, 26). In some cases no formal process may have been in place. For example, in one paper decision-makers interviewed reported that the process they used was “collaborative but unstructured” (26).

### 3.4. Criteria proposed for prioritisation of programmes for evaluations

We identified limited evidence on the use of criteria for prioritising public health programmes for evaluation, with only two papers explicitly addressing this issue (49, 51).

The paper by Ogilvie et al. recommended a framework of five questions for prioritising the evaluation of public health interventions within the context of budget constraints (49). They proposed a discussion-based approach where the questions would stimulate and structure debate amongst stakeholders such as researchers, funders, and policy makers rather than a checklist-based approach with specific criteria with formal weighting.

The proposed questions from this study are listed below along with our mapping of their concepts to our domains:

1. Where is a particular intervention situated in the evolutionary flowchart of an overall intervention program? This question aims to determine what phase the intervention is currently in, whether that be the conceptual phase, the development phase, implementation phase or full roll-out phase. We mapped this question mainly to the “feasibility” domain due to the emphasis on the effect of the phase of development of the intervention on the ability to conduct an evaluation and the type of evaluation which could be conducted. It also touched on “organisation acceptability” and “political considerations” as the study authors suggested considering the socio-political context of the intervention, as well as the degree to which it is embedded in organisational contexts.
2. How will an evaluative study of this intervention affect policy decisions? This question relates to whether there is a “customer” for the evidence generated by the evaluation, and whether it can influence policy decisions. As such, we mapped this question to “organisational/provider/industry considerations” domain, as it relates to whether the evaluation aligns with (and feeds into) the strategy and policy making of the public health organisation commissioning the evaluation. As well as considering the utility of the evaluation to decision-makers, the authors suggest considering whether the evaluation is of wider public interest, which corresponds to “social considerations.” They also specify that the type of data required from the evaluation to impact policy should be considered (for example, data relating to efficacy, effectiveness, acceptability, implementation, reach, uptake, mechanism or dissemination of an intervention, or to a cost-effectiveness/cost–benefit analysis). These factors correspond to our “health impacts of the intervention,” “social considerations” and “cost-effectiveness” domains.
3. What are the plausible sizes and distribution of the intervention’s hypothesised impacts? The authors suggest that the interventions that are most worth evaluating are those that are expected to have the largest effect on the largest number of people. However, they also suggest that small scale, novel, or untested interventions should still be evaluated if they could have important adverse effects, benefits in non-health areas, effects which may contribute to widening or narrowing health inequalities, are scalable to widespread implementation, or a promising new type of intervention. These concepts correspond to our domains of “health impacts of the intervention,” “equity/fairness/ethics/equality” and “current service provision” (the latter in terms of whether the intervention is something new that is not already provided).
4. How will the findings of an evaluative study add value to the existing scientific evidence? This question relates directly to our “evidence considerations” domain as it considers the availability of existing evidence around the intervention. There is no need to evaluate an intervention if it is already widely researched, unless it is lacking more specific contextual information such as the impact on the desired population or on non-health outcomes, the mechanism for its effects, scalability, sustainability, generalizability or distributional effects (i.e., impact on inequality). For this reason, we have also linked this question to the “feasibility” and “equity/fairness/ethics/equality” domains.
5. Is it practicable to evaluate the intervention in the time available? This question relates to our “feasibility” domain, as it discusses the practical considerations affecting whether and how an evaluation can be carried out, such as: the time needed to carry out data collection and evaluation, whether this intervention has been around long enough to see an impact, how long it might take to get results of the evaluation, whether it is possible to isolate the intervention’s impact/outcomes from other factors, and whether the context or content of the intervention changes over time, giving an opportunity to study the impact of these changes. Additionally, the authors touch on the availability of resources for evaluation, therefore relating to the “budget impact” of the evaluation.

The second paper, which touched on the prioritisation of evaluations, was a primary research paper (51) included in one of the systematic reviews (8). It carried out a process to prioritise evaluations and research within safe motherhood programmes in three developing countries: Burkina Faso, Ghana, and Indonesia. The study did not lay out specific criteria for use in prioritisation, instead utilising a structured participatory process with stakeholders to identify potential evaluation questions, their views on the important programme characteristics and other contextual information, and finally to agree on their preferred evaluation questions. We did not include it separately in the data extraction of criteria for our review, as the paper did not explicitly specify criteria for prioritisation.

### 3.5. Use and utility of the identified criteria

Overall, we found that 14 literature sources gave evidence for use or utility of the frameworks or criteria to some extent, based on actual observations, a formal assessment or anecdotal evidence (4, 10, 17, 20, 21, 23, 25, 27, 29–32, 39, 40).

#### 3.5.1. Use of frameworks/criteria sets by decision makers

Two studies asked decision makers about their use of formal priority setting frameworks or tools (20, 21). One of these assessed the extent to which formal priority setting processes were used in state health agencies in the US during times of austerity (20). One out of six of the state health agencies surveyed reported systematic use of formal priority setting frameworks. The second paper also revealed a lack of use of formal priority setting tools in England at that time (21). Although many of the decision-makers in this study described use of locally developed tools or frameworks in prioritisation they noted several limitations to these, and that “there was often a lack of transparency and systematic rigour in the prioritisation process.”

#### 3.5.2. Evidence of wider prioritisation framework use

Only the frameworks for prioritising infectious diseases from the Robert Koch Institute (RKI) and ECDC were reported as being used in multiple settings by different groups (25, 27, 31, 32). The RKI infectious disease framework was used to prioritise pathogens or was discussed in four of the included studies (25, 27, 31, 32). In one study, the RKI collected stakeholder and expert feedback on an earlier version of their prioritisation framework, in order to further refine it for the next round of prioritisation in Germany (25), which was reported in a later publication (31). Another paper applied the RKI framework in Sweden (32).

The RKI infectious disease framework, along with the ECDC infectious disease prioritisation tool designed for use in the European region (40), also influenced the approach used in a study prioritising infectious disease threats in Belgium (27). However, the approaches were significantly adapted based on context and research aims.

Additionally, the WHO-INTEGRATE framework was described in two included studies led by the same first author (10, 39). The first paper describes the development of the framework, which aimed to have wide applicability across disease areas and contexts (10). The second paper adapted the framework for the prioritisation of non-pharmacological interventions targeting COVID-19 into the WICID framework version 1.0 (39). However, neither of these papers describe usage of these frameworks in practical situations.

#### 3.5.3. Utility

This review defines the utility of a framework or set of criteria as its “usefulness” in achieving improved health and economic outcomes, or by gauging stakeholder satisfaction with the framework and criteria sets. Few studies formally addressed utility. Most of the information provided about “usefulness” was anecdotal, either from the stakeholder or study author perspective.

Five sources provided empirical or quantitative assessments or evidence of utility (17, 23, 25, 29, 30). The first assessed stakeholder views on the usefulness of the RKI pathogen prioritisation process *via* a survey (25). Respondents were largely from Germany or other EU countries (63/72 participants), and most were public health professionals, other health professionals or researchers (57/72 participants). Most stakeholders from the survey considered the prioritisation of pathogens useful for public health purposes (62/72), surveillance and epidemiological research (64/72), and for public health services at national (58/72) and international level (49/72). Fewer respondents thought it would benefit regional or local public health services (33/72 and 29/72, respectively). These views may reflect the nature of the topic, as prioritisation of infectious diseases and the public health response to these is likely to need to be led from a national/international level.

The second discussed how a pilot priority setting exercise using PBMA was used to prioritise disinvestments to address a forecasted budget deficit of $4.7 million Canadian dollars within a Canadian health authority (30). The exercise resulted in recommendation of 44 initiatives for disinvestment that had the potential to save over the deficit amount annually. Stakeholders were reported to be “extremely positive” about the process and its outputs when interviewed, finding it more robust than previous processes that were based on historical patterns of resource allocation and/or politics. They recognised that the approach could provide value in the development of investment cases and re-allocation of funds and encouraged its wider rollout within the authority.

Similarly, another study reported on a PBMA exercise implemented as part of efforts to improve resource allocation processes in Canada’s largest autonomous local public health agency (29). The study described the process as being “well received” by municipal officials and was able to generate recommendations for shifting budget towards programmes which were anticipated to provide greater health benefits. It was also reported that the transparency of the approach increased trust between the senior team and the health board, but staff working in areas recommended for disinvestment did not approve of the process.

The third study reported a mixed methods study evaluating the value and use of the Public Health England (PHE) Prioritisation Framework (23). They found that the tool was welcomed by the local authorities which adopted it and they considered it a useful tool to provide recommendations for changes in investment practises. Its systematic framework, collaborative and transparent approach were seen as beneficial. However, users noted that the process was time consuming, and that the political context for making these decisions (i.e., local government) could hinder the adoption of the tool, as elected officials make the final decisions.

Finally, one review of prioritisation processes using MCDA reported that just under half of the included studies (46%) specifically noted that MCDA was beneficial to the decision-making process (17). Similar to other studies, this review reported benefits including the systematic, transparent and flexible approach that fostered multi-disciplinary collaboration. Almost a third of studies (31%) also noted the potential for subjectivity in steps in the selection, weighting and scoring of criteria. The review did not report on the methods used for evaluating the strengths or weaknesses of MCDA in the included studies.

The potential benefits reported in these studies contrasts with other studies, which described situations in which no formal priority setting frameworks or criteria sets were in use, and the influential factors identified posed challenges (such as political, donor and industry influences; cultural and religious conflicts; and bias towards the most prominent people in society) (4).

## 4. Discussion

### 4.1. Criteria for prioritisation in public health

In this scoping review of 40 papers, we identified over 300 individual criteria used to aid decision-making in the prioritisation of public health interventions or diseases. These individual criteria were categorised into 16 high-level domains, which reflect areas that were well-reported in the literature. We found very limited evidence on the use of criteria to prioritise public health programmes for evaluation as only two studies addressed this topic specifically (49, 51).

The most frequently reported domains considered in prioritisation in public health were “burden of disease,” “social considerations” and the “health impacts of the intervention.” “Burden of disease” may be commonly included to direct resources to the diseases having the greatest impact on a population’s health. Data on burden of disease are often quantitative and likely to be routinely collected by public health organisations. Similarly, data on the health impact of an intervention are likely to be available if it is being considered for implementation; its inclusion allows targeting of resources to interventions which have the greatest benefit for the population. The “social considerations” include issues such as levels of public concern or pressure to address a disease; likely acceptability of the intervention including any social, religious, or cultural barriers to its uptake; and whether the programme would strengthen local communities and have wider social impacts. Inclusion of this domain aims to maximise the likelihood that an intervention or programme is a priority for the local community and will be acceptable and used.

The least identified domain was the “cost of disease from a patient perspective” as it was only explicitly reported in four of the 40 included studies, all of which included data from LMICs (8, 10, 16, 36). A possible explanation for this may be that the costs of public health activities often fall on the health system rather than the patient, particularly in high-income countries.

Whilst the individual cost domains were not as frequently utilised as burden of disease for example, amalgamating these (“budget impact,” “costs of disease from a health systems perspective,” “cost of disease from a patient perspective,” “cost-effectiveness,” and “societal costs of disease”) shows that cost is among the most frequently considered issues, highlighting the integral influence of finance in public health decision-making.

The infrequent explicit consideration of the patient costs did not indicate a lack of consideration of patient- or public-focused criteria. Other domains such as “social considerations” and the “costs from a societal perspective” encompassed criteria including public perception, patient demand for and uptake of the intervention, whether programmes provide tangible outcomes for the public and wider benefits for carers and families.

The frequency of domains reported in this study does not necessarily equate to their importance in the decision-making process. For instance, the domain “legal & regulatory frameworks” was only identified in 14 of the 40 included studies. However, if provision of an intervention or programme is mandated in law, or conversely if local legal and regulatory frameworks prevent its implementation, this would override other considerations. Fundamental issues such as this may be considered before programmes are entered into a prioritisation process, making them less commonly cited as explicit prioritisation criteria.

One included review also noted that the frequency of use of a particular criterion should not determine its relevance in the prioritisation process, instead this should be informed through a normative perspective (10). It reported that the value and relevance of individual domains and criteria will depend on context in terms of the topic area (for example prioritising infectious disease versus broad prioritisation across public health programmes) and the system in which the prioritisation is being carried out (10). Within prioritisation processes using approaches such as MCDA allows the individual criteria to be weighted based on stakeholders’ views on their importance.

### 4.2. Involvement of stakeholders in prioritisation processes

Involvement of a wide range of stakeholder representation in prioritisation ensures that diverse perspectives and information are captured, reducing the chance that important considerations will be missed or that there will be unintended consequences of implementing an intervention. This is particularly important for more qualitative criteria, such as “equity/fairness/ethics/equality,” “social considerations” such as local community views, and the “feasibility of implementation,” where relevant information may not be readily available and instead relies on knowledge of individuals in local communities or professionals who are providing the relevant programmes and services.

The stakeholders involved in the prioritisation of public health activities may vary according to geographical region and public health function. In Germany, public health policy shifted primarily from federal to local level in the 1990s, hence giving local public health officials greater autonomy to implement, monitor and evaluate activities based on evidence and the needs of local communities (52). The most common types of stakeholders reported as taking part in prioritisation in the included studies were policymakers, public health officials and other government officials (including local government officials in health and other departments including finance, and elected members). Other types of stakeholders involved in prioritisation included healthcare and allied professionals, health economists, statisticians, researchers, information analysts, non-governmental organisations, international donors or development partners, private sector representatives (e.g., of the pharmaceutical industry), the general public, patients and their caregivers, advocacy and civil society organisations, and ethicists.

Involvement of patient and public representatives in prioritisation of public health programmes or their evaluation is crucial, as it ensures that programmes can better reflect service user and community needs, views and priorities. Public and patient priorities and values may differ from those of public health professionals, so their involvement offers complementary perspectives and insights, leading to a more holistic approach to prioritisation. For example, patients often prioritise interventions that increase quality of life over those that lengthen life but leave the person in relatively poor health, and their choices may not align with the aim of maximising quality-adjusted life years lived (53). Public involvement also increases the level of public accountability in the prioritisation process and has the potential to increase public acceptance of prioritisation decisions and uptake of selected interventions or programmes, as well as adherence to certain measures (54).

Despite issues relating to “social considerations” being commonly considered in prioritisation, clear reporting in the studies of direct involvement of patient/public representatives in prioritisation was less common, particularly in the studies from higher income settings. One included review noted that public participation in priority setting in high income countries has not been as effective as intended, with most relying more on expert opinion (16). In cases where local government representatives are involved in public health prioritisation (as in England and the US) they may provide the perspective and preferences of local communities. Patient or public involvement was more commonly reported in a review of studies from LMICs (8). This may reflect the inclusion of studies focusing on health technology assessment, which has more established methods for patient or public consultation.

There is no consensus on the most effective way to involve the public in priority setting, but a combination of using wider consultations with more in-depth engagement have been suggested to be beneficial (30). Community participation in prioritisation exercises can be challenging due to barriers such as lack of understanding of the process, lack of time or financial resources or limited skills to meaningfully engage with other stakeholders (55). Decision-makers should work to overcome these barriers to increase public engagement with prioritisation processes.

There are opportunities for public involvement to take place within countries’ existing structures and frameworks in which research and stakeholder knowledge is transferred. For example, in Germany, Federal Health Reporting (Gesundheitsberichterstattung des Bundes, GBE) is a federally mandated system that feeds a wide array of national data and information on population health and healthcare services to the government (56). Additionally, the growing number of citizens’ forums (randomly selected citizen assemblies to address social issues) can be regularly utilised as existing vessels for the discussion of civil stakeholder preferences and priorities in public health decision-making.

Broad stakeholder involvement contributes to increasing the legitimacy, credibility, acceptability and ownership of prioritisation decisions (55). Informal prioritisation processes can allow undue influence from “powerful” stakeholders who can have a negative influence on the priority setting process and drive priorities further away from what is actually needed (4). Therefore prioritisation exercises should aim to engage all relevant stakeholders in a transparent and formal process.

### 4.3. Criteria for prioritising public health evaluations

The CDC describes public health evaluation as a “systematic method for collecting, analysing, and using data to examine the effectiveness and efficiency of programs and, as importantly, to contribute to continuous program improvement” (57). Additionally, the WHO states that the principles of public health evaluations should encompass values that can be embedded into programme descriptions in order to define its mission and acceptable standards (58). Evaluation of public health programmes is important to ensure that they are providing value for money. However, as with public health programme implementation, the existence of limited budgets means that not all programmes or even programme components can be evaluated, leading to a need for prioritisation (49).

This review found very limited evidence on prioritisation of public health programmes for evaluation, with only two studies addressing this issue (49, 51). One of these papers (51) was included in a systematic review of prioritisation in LMICs (8). It discussed the prioritisation of safe motherhood programmes for evaluation, across three LMICs, but as it focused on process rather than specific prioritisation criteria, therefore was not informative for our research question.

Conversely, the paper by Ogilvie et al. proposed five key questions which decision-makers can use to structure discussions when deciding on what evaluations should be conducted, from which we could elicit prioritisation criteria pertaining to our domains (49). Similar to the Bradford-Hill approach to assessing evidence for causation in epidemiology (59), the authors caution against treating their questions as simply a checklist of criteria. Instead, the questions are intended to structure iterative debate amongst decision-makers who can judge the weight which should be applied to each consideration, and the strength of evidence in favour of evaluation (49).

The 10 domains touched on by these questions seem a reasonable basis on which to consider prioritisation of public health programmes for evaluation, particularly in high income settings. While the authors caution against a “checklist” approach, this does not preclude use of a structured collection of data pertaining to the underlying issues/domains touched on, to inform the discussion and allow greater transparency. It does, however, support the importance of allowing a nuanced discussion of the complex issues rather than allowing a single scoring or rating system to take over.

The questions discussed by Ogilvie et al. were informed by the concept of “evaluability assessment,” which is a systematic method of determining whether a programme is ready for evaluation and how the evaluation could improve it (49). It was developed in the mid-1960s after some large-scale (and costly) evaluations of social interventions found that they were of no benefit (60). The approach is meant to be a low-cost exercise, and includes collection of information on the intervention through structured engagement with stakeholders, assessment of the available evidence, and identification of key outcomes for the evaluation through development of a theory of change, resulting in recommendations for proposed evaluation designs.

The evaluability assessment approach does not focus on comparing interventions in order to select which to evaluate, and as such most of the literature we identified did not explicitly link it to the prioritisation of programmes for evaluation (52, 60, 61). More recently an updated process has been proposed which could be applied across programmes/interventions to identify which were suitable for evaluation (62). However, even this paper focused more on feasibility of evaluation and did not explicitly link this with prioritisation of public health evaluation resources.

Ogilvie et al., however, recognised the potential of the evaluation assessment approach for prioritisation purposes when there needs to be a subset of interventions selected for evaluation because of limited financial resources. They adapted the approach to be less focused on the theory of the intervention, and more focused on practical considerations. As such, one of the key domains considered in their questions is feasibility of the evaluation. For instance, the stage of development of the programme is important as those programmes in the early stages may not have enough data or outcomes to make the evaluation useful (49).

The criteria for prioritisation of evaluation in Ogilvie et al. have some similarities to the CDC’s Framework for Program Evaluation in Public Health (1999), which cites its own four standards for evaluation of public health interventions; utility, feasibility, propriety and accuracy (63). There are clear links between the first two standards and the questions posed by Ogilvie et al. The CDC’s feasibility standard relates to the practicality, viability and pragmatism of evaluation, including the consideration of differing political interests and making the evaluation a cost-effective use of resources investment. These concepts touch on the “feasibility,” “political considerations,” “cost-effectiveness,” and “budget impact” aspects considered in Ogilvie’s questions.

The CDC’s “utility” standard ensures that information needs of evaluation users are satisfied, and addresses similar concerns to Ogilvie’s questions on the potential policy impact of the evaluation. The concepts relate mostly to “organisational/provider/industry acceptability” (in terms of understanding stakeholders’ needs) and to “evidence considerations” (in terms of presenting and disseminating the evidence generated). These domains are also touched on by Ogilvie et al. The propriety (ensures that the evaluation is ethical and legal) and accuracy (ensures the findings of the evaluation are correct) CDC standards are not closely allied with the concepts covered by Ogilvie et al.’s questions.

Whilst the criteria elicited from Ogilvie et al. covered 10 of our 16 high-level conceptual domains, some of the remaining domains could also have potential relevance when prioritising public health programmes for evaluation. The individual cost of disease domains and burden of disease were not specifically touched on by Ogilvie et al.’s five questions. They could be relevant to consider if there has been a change in disease burden or expenditure relating to the disease or intervention which suggests that existing public programmes may not be working effectively. In practise, however, such changes may not be easily (or solely) attributable to issues with programmes. Additionally, the “international support/donor acceptance” and “cost of disease from a patient perspective” domains may be more relevant to LMICs, who rely more heavily on foreign aid for healthcare and have greater out of pocket expenses for patients in comparison to HICs, who have more developed economies and can independently finance their own public health services.

The “legal and regulatory framework” domain was also not considered by Ogilvie et al. and may be less relevant in prioritising programmes for evaluation. If interventions are implemented according to their legality, then it can be assumed that they are also sanctioned to undergo evaluations. Should a legal imperative exist to carry out a specific evaluation this would override any other prioritisation criteria and the evaluation in question would not enter the prioritisation process.

### 4.4. Use and utility of frameworks and criteria sets

In terms of use, very few frameworks or criteria sets showed use beyond a single study in the included literature. Reports of decision-maker experiences showed that often formal frameworks are not utilised, and the processes are less structured (4, 21). While the complexity of public health decision-making means that it may require a more adaptive rather than prescriptive approach, there are downsides to this lack of standardisation of processes, frameworks, and tools. Informal processes lack transparency around the processes undertaken, the evidence used and the influence of stakeholders.

For example, one study reported on the experiences of those involved in priority setting for non-communicable disease (NCD) control in Kenya in the context of developing an improved model for decision-making (4). The influence of many of the factors they reported as shaping decision-making were considered to be negative, such as undue influence of donors, health professionals looking after personal interests, influence of industry, focus on needs and experience of only prominent members of society, which can lead to a shift in priorities that have been demanded rather than what is really needed (4). In reality, this is a pressing challenge for LMICs whose current health system financing relies heavily on donor support, incorporating better engagement of all stakeholders (including local-level stakeholders) may ensure the use of more transparent, robust and effective prioritisation processes.

Tools which have been developed specifically for use in prioritisation and have been reported to be utilised in practical prioritisation exercises included: the PHE Prioritisation Framework, the ECDC and the RKI infectious disease risk ranking tools, and the PAHO adapted Hanlon method (23, 25, 27, 31, 32, 40, 41). With the exception of the infectious disease tools, there were no published uses of these frameworks in different settings than those in which they were originally developed. This highlights the global (and contextual) variation in priority-setting in public health.

In contrast, prioritisation processes such as MCDA in particular have more widespread use (8, 10, 15–17, 21, 23, 27, 28, 40, 41, 43). In these approaches, as in some prioritisation tools, the criteria which should be considered are not prescribed. Instead, they focus on laying out a systematic and robust process which allow stakeholders to select the criteria which best suit their context, purpose and needs (40, 41). The flexibility of these approaches is likely to have encouraged their use, particularly given that the complexity of prioritisation does not lend itself to a one-size-fits-all set of criteria.

In terms of utility of the identified tools/frameworks for priority setting, while several papers discussed this anecdotally there were very few formal assessments. A few studies assessed economic outcomes or collected stakeholder views of the prioritisation process (17, 23, 25, 29, 30). The evidence generally suggested that prioritisation frameworks or tools can be used to make budgeting decisions including disinvestment decisions (23, 29, 30). Both the decision-makers and other experts reported that prioritisation tools or frameworks are helpful for making prioritisation decisions for public health purposes (17, 23, 25, 29, 30). The benefits were largely seen to relate to making the process more robust, systematic, transparent and collaborative (17, 23, 29, 30). Some of the drawbacks noted were that the processes can be time consuming and that political context can impact adoption of these tools (23).

None of the included studies assessed the impact of prioritisation on health or health-related outcomes. The assessment of some health outcomes may not be feasible because it would require longer-term follow up on a population-wide scale and strong data collection systems which utilise significant resources, requiring large investments. Additionally, the health outcomes of a population are unlikely to reflect the utility of the prioritisation process alone, as many other factors influence health improvement.

The paucity of assessments of priority setting approaches has also been noted by other authors, suggesting that this may be linked to limited guidance on how best to conduct these (16). There are some proposed frameworks for assessing whether priority setting is successful (16), including the accountability for reasonableness framework which looks at whether the process is legitimate and fair (64), another encompassing both process and outcome (65), and others specifically designed for LMICs (66) or PBMA processes (67). However, these do not seem to have been widely adopted. Agreed standards by which to judge the quality and outcomes of prioritisation exercises would be beneficial in encouraging robust and effective processes (68).

### 4.5. Complexity and practicalities of prioritisation

Similar to other studies, we also found that the concept of priority setting for resource allocation in public health is complex (3, 4, 10). Heterogeneity within the literature includes varying geographies, health systems, levels of prioritisation, and differences in what was being prioritised, as well as differing prioritisation processes and criteria. While prioritisation processes such as MCDA were clearly widely used, there was little evidence of widespread use of specific frameworks of criteria. Furthermore, some studies demonstrated that formal priority-setting processes or frameworks are not always used. The use of unstructured approaches to prioritisation creates a lack of transparency and consistency of how prioritisation decisions are made, hence further adding to the complexity.

For instance, economic context can play a role in shaping prioritisation processes. Our review predominantly included studies from HICs, where the burden of disease, social aspects, and the health impacts of the intervention were the most used high-level domains to inform public health prioritisation. However, this differs from LMICs. The included review which focused on public health prioritisation in LMICs found a greater emphasis on “cost-effectiveness,” the “health impacts of the intervention” and “equity” domains (8). The use of economic evidence such as cost-effectiveness in LMICs may be driven by global initiatives such as the “WHO’s Choosing Interventions that are Cost-effective” (WHO-CHOICE) tool, which provide guidance on prioritising health activities according to impact and cost-effectiveness in LMICs (8). It may also reflect the inclusion in the review of studies on areas such as prioritisation of drugs for reimbursement which are better suited to traditional cost-effectiveness analysis than the areas typically considered to fall within the remit of public health within high income settings.

Additionally, there was variability in the type of analysis or information being reported in the studies, largely falling into three categories:

- The development and/or use of a formal prioritisation tool or framework in practise.
- The development of a theoretical framework for prioritisation with no clear link to an application in practise.
- The feedback and experiences of decision-makers in past prioritisation exercises (where formal tools/frameworks/processes were not necessarily used).

In the first two categories the approach has been purposefully selected by the organisation or authors as being well suited for use in prioritisation. There were similarities in the criteria described in these two types of papers, with “burden of disease,” “health impacts of the intervention or programme,” “social considerations,” and “equity/fairness/ethics/equality” being the most cited domains in both. There were also differences. For example, “feasibility of implementation of the intervention/programme” was more commonly assessed in papers where the framework was being used or tested in practise, suggesting that these frameworks tended to have a more practical focus. These papers also tended to consider “cost-effectiveness (value for money)” more often than solely “budget impact,” which may suggest a focus on getting the most value out of a fixed budget.

In studies that collected feedback and experiences of individuals involved in priority setting, the criteria or factors identified as influencing these decisions did not necessarily represent an ideal or recommended approach. Whilst criteria relating to “burden of disease” and “social considerations” are also commonly reported in these studies, other factors such as “political considerations” are more often reported as influencing decision-making (3–5, 18–22, 24, 25). Decision makers in these studies often reported not using structured processes and frameworks, which may indicate that political considerations have more influence in informal prioritisation processes. Alternatively, it may be that political considerations, while not explicitly listed as a criterion in formal frameworks, still have an influence when they are used, either through how criteria are scored or weighted, or in qualitative discussions that can moderate the results of scoring exercises. Studies that reported decision makers’ experiences also commonly cited criteria relating to “organisational/provider/industry considerations” and “feasibility of implementation,” again suggesting a more practical focus than the papers which solely described theoretical frameworks.

### 4.6. Limitations

Our review searches were conducted in English and literature published in the English, French and German languages was eligible for inclusion. This approach may have missed some relevant studies outside of these languages, particularly those with no English language indexing and those in the grey literature. The decision to limit the included languages was made owing to time and resource constraints.

The classification of criteria into domains was carried out with the aim of showing broad areas which could be considered in a prioritisation approach. Classification was straightforward in some cases, but not in others due to either a lack of clarity in what the article authors intended by a particular criterion, or a concept having the potential to map to more than one domain based on interpretation. For example, several studies included criteria relating to various aspects of health service utilisation, which does relate to the cost of the disease to the health system, but this was not given as an explicit rationale for considering this criterion in the original studies. Instead, it tended to be framed as a measure of disease burden or threat, and therefore we have categorised as such to remain true to the study reporting. Hence, there may be some inaccuracy in the categorizations in terms of what the original authors intended, but we have aimed to avoid assumptions as to what was intended and to be as consistent in our approach as possible.

Additionally, we could only document criteria explicitly reported in the included papers. There are likely to be implicit criteria not reported in the papers which affect either the selection of areas to include in a prioritisation exercise or how the stakeholders weight the criteria or make decisions. For example, in some studies local public health stakeholders put forward possible areas for budget changes which were then prioritised, however, the reasons behind their selections (i.e., the criteria used) were not reported (29, 30). In addition to implicit criteria, many organisations may also not formally publish their approaches to prioritisation. We aimed to minimise the impact of this reporting bias through including a wide spectrum of reports on prioritisation criteria used in public health, including academic and grey literature, as well as empirical and non-empirical reports. The inclusion of papers reporting qualitative analyses of decision-makers’ experiences of prioritisation should also help to capture criteria from processes which have not been formally published or which are not explicitly listed in formal publications.

Our tallies of the frequency of use of individual domains includes both individual primary studies and reviews which include multiple studies, and this may distort the relative usage of these criteria. Some of the review studies did not quantify the frequency of criteria reported so it was not possible to disaggregate their findings.

The included studies ranged in publication dates from 2002 to 2022, and more recent papers may be more reflective of current practise and thinking in prioritisation. For example, in England, public health governance has undergone change in this period, moving under the remit of the local government from the health service in 2013. As the included paper by Marks et al. (21) describes practises pre-dating this change it reflects the situation in public health prioritisation in England prior to this move and sets the background for the development of PHE’s prioritisation framework (41), which was intended to counteract some of the problems identified.

Our grouping of criteria into high-level domains is intended to aid rapid conceptual understanding, in some instances it may result in a more generalised understanding of their meaning. Decision-makers involved in prioritisation who aim to utilise these domains to design a prioritisation framework should refer to the additional information on the definitions of each domain and the criteria they contain (in the Supplementary material) to gain a more detailed understanding of what criteria could be selected within each broader domain. The list of criteria reflects what was identified in the literature, and as such is not exhaustive but can be used as a starting point for developing context specific sets of criteria.

While this review provides a summation of the evidence identified, for any given context, more in-depth examination of those included papers which reflect a similar context to that of the stakeholders/decision-makers may provide additional insight to identify the most appropriate criteria for inclusion.

## 5. Conclusion

Overall, while much has been written about prioritisation in healthcare broadly, relatively few studies have assessed prioritisation in public health specifically, and there is little evidence on the prioritisation of public health evaluations. Additionally, there has been very limited formal evaluation of the use and utility of these prioritisation methods.

Our review compiled a broad range of criteria which have been used or proposed for use in public health prioritisation in the existing literature and classified them into domains. These findings aim to inform the discussion on which of these criteria and domains are best suited to the prioritisation of public health programmes to be evaluated, and development of a framework for this purpose. Such a framework would fill an evidence gap, as would formal assessment of its utility.

As per the approaches used in many prioritisation studies, selection of prioritisation criteria is best carried out by relevant stakeholders and considering the prioritisation context. To ensure prioritisation reflects the needs of all involved in or impacted by the intervention or evaluation, a broad stakeholder involvement is necessary in the prioritisation exercise, including public participation.

## Data availability statement

The original contributions presented in the study are included in the article/Supplementary material, further inquiries can be directed to the corresponding author.

## Author contributions

AB carried out searches and first pass appraisal. JS and SA carried out second pass appraisal of the search, full text appraisals, citation tracking and reference harvesting, data extraction from the included studies and categorisation of criteria identified in the studies into domains. AW carried out duplicate appraisal of studies that reached full text appraisal, and duplicate extraction of study criteria and categorisation into domains. Any disagreements were resolved through discussion with BB and CEB as needed. SA and JS drafted the review. AW checked and edited the draft. BB reviewed and provided comments on the retrieved literature and the draft manuscript. CEB designed the research question, provided overall guidance to the research, reviewed, and commented on the draft manuscript. All authors approved the final manuscript.

## Funding

The Robert Koch Institute in Germany provided funding to conduct the research and for the open access publication fees.

## Conflict of interest

The authors declare that the research was conducted in the absence of any commercial or financial relationships that could be construed as a potential conflict of interest.

## Publisher’s note

All claims expressed in this article are solely those of the authors and do not necessarily represent those of their affiliated organizations, or those of the publisher, the editors and the reviewers. Any product that may be evaluated in this article, or claim that may be made by its manufacturer, is not guaranteed or endorsed by the publisher.
