# Supplementary material for: Prioritisation processes for programme implementation and evaluation in public health: A scoping review
Source: Front Public Health. 2023 Mar 27;11:1106163. doi: 10.3389/fpubh.2023.1106163 (PMC10083497; doi:10.3389/fpubh.2023.1106163)
Supplement: Supplementary file 1 [file Data_Sheet_1.docx]

Supplementary Material

## 1 Review Protocol: Prioritisation in public health

**Context**

Prioritisation in public health investment considers 1) dynamics of accountability triangulation between health, healthcare, and politics, 2) broader priority-setting within local authorities, 3) perceptions of evidence, and 4) classification of interventions (Marks L. et al., 2015). Developing priority rating systems for public health is not a new dilemma. In 1988, the CDC published its guidelines for setting priorities in public health (Centers for Disease Control and Prevention, 1988) and Vilnius and Dandoy dealt with the topic as early as the 90s by developing a model that ranks public health issues according to size, urgency, severity of the problem, economic loss, impact on others, effectiveness, propriety, economics, acceptability, legality of solutions, and availability of resources (Vilnius D, 1990). Epidemiology is one domain in public health that can be used to set up priorities. For instance, decision makers can consider burden of disease or epidemiological indicators collected in most surveillance systems when prioritizing health areas to target when faced with the choice. With the multiplicity and complexity of available decision-making models, some health departments have developed epidemiology-based digital applications to help prioritize scarce public health resources (Simoes E et al., 2006). However, quantitative methods are not the sole basis for prioritizing health domains. Shopper et al demonstrated more than 20 years ago that a mix of quantitative and qualitative research methods is needed when prioritizing strategies for public health prevention (Schopper D et al., 2000).

While evaluation should be an integral part to every public health program, resource limitation can lead decision-makers and program managers to dedicate all available resources to implementing the programs themselves. RKI aims prioritise conducting evaluations in order to use resources in the most efficient, yet useful way. However, while the literature offers a variety of rating systems and guidance for prioritization of public health activities, and while these rely on an even more varying set of criteria, no prioritization process exists specifically for choosing public health interventions, programs, or activities for evaluation. Hence, there is a need to systematically map and review the evidence on criteria that can be used to prioritize public health programs for evaluation. This review will be used to inform RKIs development of a framework for prioritization of evaluations.

**Review questions**

1. What criteria have been proposed for prioritisation of public health interventions?
2. Which identified criteria have been suggested for use specifically to prioritise public health programmes for evaluation?
3. What is the evidence on use and utility of said criteria?

The broader aim of the study is to identify the processes or criteria that can be used to prioritize public health programmes for evaluation.

**Searches**

The search strategy will focus on review question 1 (RQ1), with information relevant to all three review questions extracted from the identified studies.

The search will include broad concepts encompassing priority setting in public health. Initial scoping searches will be carried out to test different search approaches and search strings, and the yield of different databases (MEDLINE (PubMed), EMBASE and The Cochrane Library). Based on the findings of these scoping searches the final search approach will be developed.

To expand the search results, we will undertake manual reference harvesting from the bibliographies of relevant papers and also conduct citation tracking of key papers.

Key websites of international, regional and national public health bodies will be searched including:

- WHO
- CDC
- ECDC
- International Association of National Public Health Institutes (IANPHI).

Google scholar searches will also be used to identify relevant grey literature.

Searches will be conducted in English language and cover the period 2007 to date. Studies written in English, French, and German will be considered for inclusion.

All references will be managed through EndNote v.20.

**Types of study to be included**

Due to the descriptive nature of the questions (particularly Q1 & 2), no study design criteria will be applied. We will include all study designs as well as recommendation papers on public health prioritisation criteria and frameworks from key international and national public health bodies, regardless of whether they are described as part of an empirical research study or not for RQ1, 2, and the “use” aspect of RQ3. For the “utility” aspect of RQ3 only empirical research studies will be included.

**Condition or domain being studied**

Prioritisation in public health; Decision making.

**Participants/population**

Studies from any country will be included.

**Intervention(s), exposure(s)**

We will include papers describing any criteria or frameworks used to prioritise public health areas or public health programs e.g. for investment, other resource allocation, or policy making.

Definition of prioritisation: *“Prioritisation is a process whereby an individual or group places a number of items in rank order based on their perceived or measured importance or significance”* (CDC,1991).

**Public health** covers a range of functions including disease prevention, health protection, health promotion and health improvement. What falls under the remit of public health may vary according to geographic setting. We propose to include papers which describe their focus as public health, regardless of the definition used. As the aim of this project is to support RKI in the development of their evaluation prioritization process, greater emphasis will be placed on papers which pertain to public health areas which are applicable to RKI’s remit and from geographies with similar public health functions.

Definition of public health: *“Public health refers to all organized measures (whether public or private) to prevent disease, promote health, and prolong life among the population as a whole. Its activities aim to provide conditions in which people can be healthy and focus on entire populations, not on individual patients or diseases. Thus, public health is concerned with the total system and not only the eradication of a particular disease”* (WHO, 2011).

**Comparator(s)/control**

Studies will not be required to have a comparator or control group.

**Outcomes**

***Main outcome(s)***

RQ1: The identification of the criteria used in prioritisation (RQ1).

***Additional outcome(s)***

RQ2: Information about any linking of the identified criteria or frameworks with prioritising the evaluation of public health interventions

RQ3: Information on the use of the identified criteria or frameworks in practice (e.g. which organisations or bodies have used them)

Information on the utility of criteria for evaluations.

For the utility of prioritisation framework we anticipate that measured outcomes may include e.g.

- epidemiological measures (e.g. prevalence or incidence of a behaviour, risk factor, or disease)
- economic measures (cost, other resource use cost-benefit, cost-effectiveness)
- provider-reported measures (e.g. experience using the criteria/framework)
- service user-reported measures (e.g. overall satisfaction with programmes)

We will not exclude studies based on outcomes.

**Detailed inclusion/exclusion criteria**

The following table will be updated to record any decisions made during the sifting process

| **Inclusion** | **Exclusion** |
| --- | --- |
| - Include papers on prioritisation in public health specifically (we will apply this criterion based on the study’s own description of the topic area as “public health”) - Include all countries - Include all study designs and grey literature - Include papers with single or multiple criteria/domains for prioritisation - Include papers published in English, French, or German language | - Exclude papers on prioritisation in healthcare more broadly (ie outside of government-funded public health (e.g. prioritisation within UHC, employer/workplace safety delivered programs, guideline development) - Exclude papers that describe how to conduct an evaluation - Exclude papers published prior to 2007. |

**Data extraction (selection and coding)**

Sifting will take place in three stages:

- A first pass sift at title and abstract level by an information specialist to remove clearly non-relevant material and duplicates
- A second pass sift at title and abstract level by reviewer(s) to assess relevance to the scope, this stage will err on the side of caution for potentially relevant papers
- A third pass sift stage at full text by reviewer(s) to decide on final inclusions and exclusions.

For any studies where there is uncertainty about whether to include or exclude, these will be discussed internally by the project team, with discussion with RKI if the uncertainty cannot be resolved internally. Inclusion/exclusion criteria will be clearly documented.

A PRISMA flow diagram will be used to report the study selection process.

Data will be extracted from the selected studies and populated into a characteristics table highlighting key information such as: authors, publication year, the objectives, country, type of PH programme, study design, key criterion/domains, type of analysis, and outcomes.

**Risk of bias (quality) assessment**

Quality appraisal tools for empirical studies will be selected once relevant studies have been identified.

**Strategy for data synthesis**

The results will undergo data extraction which will be presented in a tabular form. Additionally, results will be synthesized descriptively.

**Analysis of subgroups or subsets**

While the review will not exclude studies from e.g. low income countries where the remit of public health may differ, the review will focus on those studies and findings which are most relevant for the RKI context and remit.

**Review team members**

Dr Rob Cook – review oversight

Dr Alicia White – reviewing, quality assessment

Shaileen Atwal - reviewing

Anelia Boshnakova and team – literature searching

**Type and method of review**

Service delivery, Systematic review

**Bibliography**

Centers for Disease Control and Prevention, 1988. Program management: a guide for establishing public health priorities, Atlanta, GA: U.S. Department of Health & Human Services.

Centers for Disease Control and Prevention, 1991. Assessment Protocol for Excellence in Public Health (APEXPH), Washington DC: U.S. Department of Health & Human Services.

Centers for Disease Control and Prevention, 1999. Framework for Program Evaluation in Public Health. Morbidity and Mortality Weekly Report, 48(RR-11).

Marks L. et al., 2015. The return of public health to local government in England: changing the parameters of the public health prioritization debate?. Public Health, 122(9), pp. 1194-1203.

Schopper D et al., 2000. Setting health priorities in a Swiss canton: what do different methods tell us?. Epidemiology & Community Health, Volume 54, pp. 388-393.

Simoes E et al., 2006. Prioritization MICA: A Web-based Application to Prioritize Public Health Resources. Public Health Management & Practice, 12(2), pp. 161-169.

Vilnius D, D. S., 1990. A priority rating system for public health programs. Public Health Reports, 105(5), pp. 463-470.

World Health Organisation, 2011. Glossary of globalization, trade and health terms. Geneva, World Health Organization

**2 Search Strategy**

**Final search strategy MEDLINE (via OVID)**

Search date 11 March 2002

1. exp *Public Health/ 2008128
2. exp *Health Promotion/ 54869
3. ("public health" or "health promotion").ab,ti. 311150
4. 1 or 2 or 3 2261239
5. "priorit*".ti. 19511
6. (prioriti?ation or priority-setting or "priority setting" or "setting priorities" or "priority rating").ab. 14961
7. *Health Priorities/ 5639
8. 5 or 6 or 7 33371
9. 4 and 8 8101
10. (criteri* or method or framework or tool or approach* or guidance or model or process or

"decision-making").ab,ti. 7951129

1. 9 and 10 4097
2. 11 4097
3. limit 12 to yr="2007 - 2022" 3415
4. 13 3415
5. limit 14 to humans 2706

Initially the search was limited to 15 years (2007-2022) but after internal discussion this was expanded back to 2002 on 21 March 2022. This brought the total number of database search results to 2,974.

**3 Data extraction tables**

**Supplementary table 1: Data extraction table for included studies and their criteria**

| **First author** | **Year** | **Title** | **Objectives** | **Organisation carrying out prioritisation/ proposing the tool** | **Country(ies) /region(s)** | **Level at which prioritisation was carried out** | **Empirical study(Y/N)** | **Study design** | **Unit prioritised** | **Type of PH interventions/ diseases prioritised (specific/ generic)** | **Framework used (if named)** | **Criteria** | **High level domains** | **Approach/ process used in prioritisation** | **Were criteria weighted? (Y/N)** | **Actors/ organisations involved in the prioritisation process** | | **Theoretical or actual application?** | |
| --- | --- | --- | --- | --- | --- | --- | --- | --- | --- | --- | --- | --- | --- | --- | --- | --- | --- | --- | --- |
| **Systematic reviews and rapid evidence reviews** | | | | | | | | | | | | | | | | | | |  |
| Jimenez-Soto | 2012 | Prioritisation of investments in reproductive, women’s and children’s health: Evidence-based recommendations for low and middle income countries in Asia and the Pacific – a subnational focus | To review the literature and provide evidence-based recommendations to inform decisions by subnational authorities in the Asia-Pacific region on how to prioritise service delivery investments in reproductive, maternal, neonatal and child health. | N/A | LMIC in APAC | Meso (main focus) and macro | Y | Systematic review (and recommendations based on findings) | Programmes/interventions | Specific | The review mainly discussed approaches/processes rather than frameworks | Criteria suggested for consideration in priority setting for RMNCH were: health outcome (mortality, nutrition, sexual and reproductive health), intervention coverage (e.g. type and number of visits, quality of care), equity, costs (including cost effectiveness analysis, total/per capita costs, recurrent and capital costs), affordability, system strengthening and assessment of which services are delivered in the private sector, feasibility, community acceptability (including out of pocket costs), political economy | 2,3,4,6,7,8,9,11,13,14 | PBMA, A4R, MCDA, Life Saved Tools (LiST), Business Case, Investment Case, balance sheet, The Child Health and Nutrition Research Initiative (CHNRI) methodology | N | N/A | Theoretical | | |
| Kaur | 2019 | Criteria Used for Priority-Setting for Public HealthResource Allocation in Low- and Middle-Income Countries: A Systematic Review | To identify criteria being used for priority setting for public health resource allocation decisions in low- and middle-income countries. | N/A | LMIC | Macro, meso and micro | Y | Systematic review | Interventions | Generic | Not specified in most cases, except in one study where the TELOS framework was used. | Severity of disease, number affected/burden of disease, equity, cost-effectiveness, poverty reduction, budget impact, cost of care, affordability from patient perspective, health benefits, fairness/ethics/equality, social implications, provider acceptability, legal and regulatory framework conducive for implementation, political considerations, international support/donor acceptance, feasibility of implementation, influence on decision making (gender participation in decision-making, ethnicity, education/wealth/community views) | 1,2,3,4,5,6,7,8,9,10,11,12,13,16 | Varied: multi-criteria decision analysis (MCDA), accountability for reasonableness (AFR), health technology assessment (HTA), balance sheet, TELOS (technical, economic,  legal, operational, and scheduling) framework, Cookson and Dolan approach | Mixed (Y/N for different studies) | Studies included reported various actors, with policy makers making up majority of stakeholders (81%), health professionals (42%), general population (26%), patients and their organisations (21%), researchers (19%), civil society representatives (14%), reimbursement managers (9%), pharmaceutical representatives (7%), international donor support representatives (5%), and ethicists (2%). | Unclear (details of the included studies not reported) | | |
| Zhao | 2022 | A Rapid Literature Review of Multi-Criteria Decision Support Methods in the Context of One Health for All-Hazards Threat Prioritisation | To identify key considerations for the application of MCDA to prioritise all-hazard threat prioritisation from a One Health perspective by examining how MCDA has been used in health-related studies in the human, animal, and environmental fields. | Public Health Agency of Canada | OECD countries (ie included studies) | Macro and meso | Y | Systematic review (rapid) | Mixed (health risks or interventions) | Generic | Not specified (all MCDA based) | **Threat:** probability of introduction, pathogenicity, transmission dynamics, evolutionary potential, capacity to detect the threat; Health: incidence/prevalence, health impacts, population vulnerability, likelihood of increased burden; Intervention: options, complexity, sustainability, effectiveness, safety, availability of evidence; **Economic:** cost, health care utilisation, job creation potential, trade impact, industry burden; **Societal:** public awareness/concern, acceptability, disruption potential, benefit potential, social equity, community engagement; **Strategic:** impact to credibility, alignment with other initiatives, organisational capacity, impact of climate change, stakeholder pressures, political context | 1,2,4,7,8,9,11, 13,15,16 | MCDA (53/54 studies) and 1 Delphi study | Y | The most common stakeholder groups engaged included government (e.g., ministry/department reps, policy analysts etc.), non-governmental groups (e.g., advocacy groups, industry etc.), subject matter experts (e.g., economists, epidemiologists, doctors, nurses etc.) and the public (e.g., the general public, students, farmers etc.) | Not specified but appears to be mixed (ie some studies reported on real-world usages but others were conceptual/theoretical) | | |
| Stratil | 2020(a) | Development of the WHO-INTEGRATE evidence-to-decision framework: an overview of systematic reviews of decision criteria for health decision making | To provide a comprehensive overview of criteria used in or proposed for real-world decision-making processes, including guideline development, health technology assessment, resource allocation and others, to inform the development of a new evidence-to-decision (EtD) framework suitable for decision making on public health, health system and clinical practice interventions. | WHO | International | Macro, meso and micro | Y | Overview of systematic reviews | Any | Generic | Not reported | The overview identified 1 meta-criterion and 7 substantive criteria, the latter of which were made up of 45 sub-criteria and 200 'unique decision aspects'. Health related balance of benefits and harms (general considerations surrounding benefit/effect, type and composition of effect/benefit/impact (e.g. impact on mortality, disability, QoL), character of benefit or effect (e.g. timing of onset of benefit, duration of benefit), individual and population level of benefit, balance of benefits and harms, general considerations surrounding harm/risk, health-related need and priority (e.g. burden and impact of disease, size of affected population, severity of disease)), human and individual rights, acceptability considerations (perceived priority of the problem, acceptability by beneficiaries, acceptability by those providing the intervention, social and cultural acceptability; stakeholder demand, interests and pressures (e.g. from advocacy groups, industry, and those providing the intervention)); societal considerations (societal needs and priority, social and societal impact, impact on economy, innovativeness, environmental impact, impact on future generations), considerations of equity, equality and fairness (equity and equality, accessibility (including risk of catastrophic costs and cost and financial impact on beneficiaries), availability (including availability/lack of suitable alternatives, limitations of alternative interventions, unmet needs), responsibility, non-discrimination, consideration regarding specific populations), cost and financial considerations (financial burden of disease on the health system, cost and budget impact of intervention, relation of costs and benefits, financial context (appropriateness), financial feasibility (e.g. affordability), financial sustainability), feasibility and health system considerations (health-system related needs and priority, feasibility and capacity to implement, considerations of management and organisation of health system, resource considerations, considerations of human resources and their skills, considerations of non-financial physical resources (equipment, infrastructure), interaction with and impact on health system, appropriateness within health system, legislative and regulatory considerations, political considerations (including donor and global interests and pressures), strategic considerations, characteristics of intervention), evidence considerations (considered by the authors to be a meta-criterion i.e. applicable to all of the other criteria) | 1,2,3,4,5,6,7,8,9,10,11,12,13,14,15,16 | Included reviews described various approaches including e.g. MCDA, HTA | Not reported for individual reviews | Not reported for individual reviews | Theoretical | | |
| **Mixed methods studies** | | | | | | | | | | | | | | | | | | |  |
| Otim | 2014 | Priority setting in Indigenous health: assessing priority setting process and criteria that should guide the health system to improve Indigenous Australian health | To elicit the perceptions of Indigenous and non-Indigenous decision-makers on several issues related to priority setting in Indigenous-specific health care services, in particular to identify the criteria used to set priorities and to identify how the priority setting process could be improved. | Department of Human Services and Aboriginal Community Controlled Health Services, Victoria | Australia | Meso and micro | Y | Mixed methods | Programmes/interventions | Specific | None reported | *Criteria reported as being used included:* Size of the health problem, feasibility/sustainability, equity, political 'hot spots', acceptability, access to services, historical trends/ patterns, efficiency (comparison of the relationship between resource use and outcomes between different options). Frequency of use of the criteria varied between the respondents and there was no clear single set of criteria or approach used. | 1,2,3,8,11,13,14 | Not described | Not described | Directors and senior executives from the Department of Human Services (DHS, Victoria; i.e. funders of the services), CEOs and their representatives (deputy CEOs, senior executives, managers) from Aboriginal Community Controlled Health Services (ACCHS, indigenous primary healthcare service providers) were surveyed. | Actual (experiences of decision-makers) | | |
| Leider | 2014 | Budget- and priority-setting criteria at state health agencies in times of austerity: a mixed-methods study | To examine critical budget and priority criteria for state health agencies to identify likely decision-making factors, pressures, and opportunities in times of austerity | State health authorities | USA | Meso | Y | Mixed methods | Programme/services | Generic | None reported | **Top tier (most cited and identified as most important) criteria**: political interests, magnitude of the problem, is it a mandatory match or mandated service, mission critical services, seriousness of consequences of the problem, delivery by others, meets federal priorities/guidance, previous allocation and results, availability of funds, prevention and protection potential, impact on locals and partners. **Second tier criteria**: unsustainable/crippling cuts, impact on personnel, maximise/leverage dollars for other programs, emergent issues, affects a targeted group, aligned with strategic plan, affects technical expertise/capacity, cost effectiveness, means/feasibility of correcting, strength of evidence, community interests, immediate benefit, equity promoting, population based, new or expanding program vs maintaining effort, special interest groups involved **Other criteria:** non-health benefits (eg impact on work/school attendance), trend in problem (worsening/improving) | 1,2,3,4,7,8,9,10,11,13,14,15,16 | Not described | Not described | Executive leadership (CEOs, CFOs, senior deputies), legislative liaisons, and division or bureau leadership (directors) within environmental health, preparedness and maternal and child heath divisions from state health authorities (SHAs) were surveyed. In Leider 2013* these respondents reported that during resource allocation/prioritisation they quite often or very often consulted: staff/personnel (87%), colleagues in local health departments (47%), the board of health (20%), legislators (23%), the governor or governor's office (36%), or the community/public (28%). | Actual (decision-maker experiences) | | |
| Baum | 2011 | Resource allocation in public health practice: a national survey of local public health officials | To gain an empirical understanding of the types of allocation decisions local health officials (LHOs) make and the factors that influence those allocation decision | Local public health bodies in North Carolina | USA | Micro and meso | Y | Cross sectional | Interventions/programmes | Generic | None reported | Effectiveness of the activity, previous allocations, being the sole provider of the activity in the community, reluctance to lay off employees, influence from the board of health, input from staff, government guidelines, needs assessment, input from the country council, public expectations, economic analyses (e.g. cost-effectiveness analyses, PBMA), input from state, direct public input, decision tools, consulting colleagues. (Reported in order of frequency of the factor being reported as being "very influential") | 1,3,4,7,8,9,11,14 | Various: staff consultation, reviewing government guidelines, consulting the board of health, consulting other local health departments, using economic analyses, conducting needs assessment, consulting colleagues at state level, and using decision tools. (Options listed from high to low frequency of use "always" or "usually". Processes were not mutually exclusive.) | Y | Local public health officials | Actual (experiences of decision-makers) | | |
| Hunter | 2019 | Shifting the gravity of spending - mark 2: final evaluation report | **Primary objective:** To assess how the PHE PF has been adopted by decision-makers. **Secondary objective:** To analyse the economic impact of the decisions where possible | 3 local authorities in England utilising the PHE PH | England | Micro | Y | Mixed methods | Programme areas | Generic | PHE's Prioritisation Framework | Criteria used by the 3 LAs included (not all criteria used by all LAs, similar criteria have been merged): - **Local need** (size of population affected, focusing on need that was strategically aligned to existing objectives). - **health inequalities** (aim to close the gap between the best and poorest health and wellbeing e.g. in healthy life expectancy between the national average and the county). - **evidence/scale of impact/ effectiveness** (what proportion of residents would be expected to benefit from the programme, to what extent does the programme area get the results intended, the quality of evidence of effectiveness and the theoretical underpinnings of programmes as well as evidence in prevention). - **current investment** (the current budget allocation to the programme) - **value for money/cost-effectiveness** (the costs of the programme area compared with benefits and cost savings, extent to which the programme provides value for money). - **system benefits/ interdependencies/ connectedness/linkages** (focus on the impact and level of connectedness between programmes and other services). - **building community strengths** (assessment of how community-centred the programme was and the extent to which the programme strengthens the community to support PH outcomes). - **public health responsibilities** (focus on the degree to which the programme was a mandated function or contributed to a statutory LA function such as safeguarding) - **deliverable/feasible/acceptable** (the extent to which a programme is politically acceptable, technically possible, legal, environmentally sustainable, socially acceptable, and the workforce and market capacity exist or can be developed. possibility of reaching the potential for the programme area). - **innovation** (the extent to which a programme has the flexibility to embrace opportunities to improve current practice thereby better meeting identified local need) - **focus on prevention** (whether it focuses on early intervention and prevention) - p**olicy/ strategy/mandate** (local or national policy alignment and national mandates) | 1,2,3,4,7,8,9,10,11,13,14, 16 | MCDA | Y | Interviews were held with a selection of stakeholders from 3 early adopter local authorities *(public health department members including eg directors or deputy directors, PH consultants or project leads, information analysts; elected members of the LA [councillors], members of the finance department, regional PHE managers - unclear whether the latter would routinely take part in these decisions or whether this was only due to it being in the implementation phase)* | Actual (decision-maker experiences) | | |
| Kapiriri | 2022 | Priority setting and equity in *COVID-19* pandemic plans: a comparative analysis of 18 African countries | To assess the degree to which national covid-19 preparedness and response plans across 18 African countries incorporated priority setting concepts, and whether they adhere to established quality indicators of effective priority setting | Various African governments | 18 African countries | Macro | Y | Mixed methods | Interventions (covid-19) | Specific | Mixed | Explicit criteria reported to be stated in the plans: emerging nature of the pandemic, flexibility to adjust based on pandemic evolution, adaptability, disease risk, disease transmissibility, disease severity, disease burden, population density, capacity to respond, people's culture and background, equity, vulnerable patient groups, service continuity (sustenance of routine essential services), geographically vulnerable regions. Other implicit priority setting criteria: political will; financial, material and human resource availability/allocation (including budget estimates); stakeholder participation (intersectoral, ministerial, international development partners, technical experts), use of evidence, reflection of public values, mechanisms for appealing the decision, mechanisms for enforcement (e.g. supporting pandemic governance, coordination and accountability). | 1,2,4,8,9,11,12,13,14,16 | Not described in the plans | Not described in the plans | Many plans reported the involvement of inter-sectoral committees led by the Ministries of Health and including e.g. agriculture, environment, trade and industry, education, information, finance, national police, religious and traditional organisations, international development partners, the private sector, and occasionally universities. | Actual (policy analysis) | | |
| Bekemeier | 2013 | Local public health resource allocation: limited choices and strategic decisions | To identify factors influencing local resource allocation and programmatic decisions among public health leaders facing severe funding losses. | Washington State's public health practice-based research network | USA | Micro | Y | Mixed method | Not specified (areas for funding cuts) | Generic | None reported | **Factors influencing decision making:** legal mandates, availability of financial resources; workforce capacity; local policymakers and boards of health influence; community need, and availability and capacity of other community agency or partner to deliver the service; organisational history and philosophy; partnerships; availability of research evidence and data | 1,4,9,10,11,13,14,16 | Not described | N (no explicit scoring or weighting of criteria was reported, but respondents did report that some criteria were more important than others) | Washington state local health jurisdiction public health leaders (directors or lead administrators) took part in the study. Other stakeholders may take part in the actual resource allocation decision-making. | Actual (experiences of decision-makers) | | |
| Gilsdorf | 2011 | Prioritisation of infectious diseases in public health: feedback on the prioritisation methodology, 15 July 2008 to 15 January 2009 | To obtain expert feedback on the RKI's pathogen prioritisation methodology from 2004 in order to further refine it, and discuss the potential implications for the planned modifications. | RKI | Germany | Macro | Y | Cross sectional | Diseases (pathogens) | Specific | RKI pathogen prioritisation framework | Existing criteria in the RKI 2004 framework: **Burden of disease:** Incidence, severity, mortality **Epidemiological dynamic:** outbreak potential, trend, emerging potential **Information need:** evidence for risk factors/groups, validity of epidemiologic information, international duties and public attention, evidence for pathogenesis **Health gain opportunity:** preventability, treatability. Other criteria suggested or mentioned as needing consideration were: Economic impact of disease, impact on work and school absence for those who are ill or for their carers, economic impact of control measures, life years saved or lost, emergence of antimicrobial resistance, and monitoring of vaccination effects on e.g. incidence or pathogenicity, political attention. | 1,4,5,7,8,11,12,13,15,16 | RKI pathogen prioritisation process | Y | There was an open call for feedback, and additional stakeholders invited to complete the survey were: all German regional epidemiologists, all members and alternates of the scientific advisory forum of the European Centre for Disease Prevention and Control, all heads of the German national reference laboratories, all member of the Committee for Epidemiology of Infections, relevant German epidemiological societies and associations. Respondents suggested that stakeholders who should take part in prioritisation included: national public health service, university faculty of infectious diseases, microbiologists, hospital epidemiologists or hygienists, international public health organisations, regional public health service, hospital physician and local public health service, and health economists (listed in order of frequency of being suggested). | Actual (experiences/views of stakeholders) | | |
| Platonova | 2010 | Local health department priority setting: an exploratory study. | To examine the extent to which data-driven objective criteria were considered important to **public health** officials in North Carolina and the extent to which they chose between objective and subjective criteria in establishing public health priorities. | Local public health bodies in North Carolina | USA | Micro | Y | Mixed methods | Not specified | Generic | None reported | **Objective criteria**: magnitude of the problem, cost-effectiveness, trend direction (of the problem), magnitude of difference to like counties, funds availability, external directives (mandates, laws or local ordinances) **Subjective criteria**: seriousness of consequences of the issue (e.g. severity of illness, premature mortality), feasibility of correcting the problem, community acceptability, prevention potential (evidence of effectiveness), political pressure. | 1,3,4,7,8,10,11,13 | Not described | Unclear (no report of whether formal scoring and weighting was used, but participants did rank criteria in terms of their perceived level of importance) | North Carolina local public health unit directors and their representatives took part in the survey, they may not represent the only stakeholders who take part in actual decision making. | Actual (experiences of decision-makers) | | |
| Marks | 2013 | Prioritising investment in public health and health equity: what can commissioners do? | To explore commissioners' views on prioritising for investment in public health in England. This study reviewed: methods for decision support; their relevance for prioritising health and health equity in principle; and their adoption by decision makers in practice | English primary care trusts | England | Micro | Y | Mixed methods | Not specified | Generic | None reported | The areas reported as being taken into consideration included: burden of illness (including prevalence, preventable illness and disability over time); cost of illness (economic burden of risk factors on health services and other sectors; predicted service use); health inequalities (distribution of needs by economic group, ethnic group, and area; interventions needed to narrow gaps; social equity); cost-effectiveness or cost-utility of existing treatments (eg cost per QALY, impact on health outcomes and use of services of investment, return on investment); existing and desired service provision; health benefits; costs of interventions; intersectoral impacts; ethical considerations; national and local targets; alignment with strategic and financial priorities; public involvement in needs assessment | 1,2,3,4,5,7,8,9,11,14 | Mixed, including for example: programme budgeting compared to outcomes achieved, PBMA, Delphi, scenario planning, MCDA, conjoint analysis/discrete choice experiments, paired comparison, population cost-impact approach | Mixed | PCT chief executives, board chairs, directors of commissioning, directors of finance, directors of public health, general practitioners with a commissioning role and Professional Executive Committee Chairs. While no members of the public were interviewed in this process, in some (but not all) cases it was reported that the public was involved in the needs assessment process | Actual (experiences of decision-makers) | | |
| **Qualitative studies** | | | | | | | | | | | | | | | | | | |  |
| Wanjau | 2020 | Stakeholder perceptions of current practices and challenges in priority setting for non-communicable disease control in Kenya: a qualitative study | To explore the stakeholders’ perceptions of current practices and challenges in priority setting for non-communicable disease (NCD) control in Kenya. | N/A | Kenya | Macro | Y | Qualitative | Not specified | Specific | None reported | Criteria identified as playing a role in decision-making were: external factors (donor funding, influence from external stakeholders such as international bodies), national leadership factors (political influence, ministry of health policies and plans, available financing for non-communicable disease control), burden of disease (presence of disasters, greater focus on the treatment of NCDs than prevention in part due to industry), technical factors (health professionals' participation in the priority setting process, stakeholder engagement, media, data from surveillance and research), people and equity factors (experiences and needs of the prominent people in society, religious and cultural influences, influences from the general public) | 1,4,8,9,11,12 | Not described | N/A | Stakeholders who took part in the study were those involved in selecting PH interventions to implement (prioritisation), as well as those looking at preventive and early interventions strategies for NCD, or focusing on NCDs of risk factors for NCDs: MoH heads of relevant units, divisions and directorates (e.g. relating to NCDs, health promotion, standards and quality assurance), members of relevant health advisory committees, other MoH officials, representatives from other relevant Kenyan health agencies/economic bodies, representatives from other 'influential credible bodies', representatives from civil society, representatives from the Medical Research Council, officers from the treasury who interacted with the health budget, a health counterpart from the Ministry of Planning, Education, Science and Technology, representatives of relevant external partners (e.g. WHO), relevant academic experts. However, the respondents noted that stakeholder engagement in actual priority-setting processes was limited, and where it did take place it often only involved those at the top levels of management. | Actual (experiences of decision-makers) | | |
| Frew | 2019 | Health economics methods for public health resource allocation: a qualitative interview study of decision makers from an English local authority. | To better understand the context for public health decision-making in one local authority, and how economics evidence is being used; and to obtain decision-maker suggestions for how methods could be improved to better support local public health | Local authority in England | England | Micro | Y | Qualitative | Not specified | Generic | One framework mentioned: Portsmouth prioritisation tool. | Budget impact, cost of the condition (to the health system), cost-effectiveness (best outcomes for investment), evidence-base, internal organisational (Council) priorities, key performance indicators and benchmarking (local and national level, using eg the public health outcomes framework), equity (meeting diverse population need, ensuring equitable access), effectiveness, achieving outcomes beyond health (eg school readiness from early years programmes, ability to re-enter employment after illness), alignment with wider Council strategy, impact on mental health and wellbeing, patient experience, political context (including Council political time cycle), feasibility issues, service provider reaction, media reaction and public scrutiny | 1,2,3,4,5,7,8,9,11,13,16 | Not described | N (but is was discussed which ones were more important/had more weight) | Local authority stakeholders with a substantial role in decision making for budget allocation took part in the study (public health, finance, legal and commissioning officers, and elected members). | Actual (experiences of decision-makers) | | |
| Greaves | 2017 | Health and wellbeing boards: public health decision making bodies or political pawns? | To identify how public health decisions are made in Health and Wellbeing boards in England, including whether and how criteria and processes are used. | Health and Wellbeing boards | England | Micro | Y | Qualitative | Interventions (or population groups) | Generic | None reported | Consistency with mandate/strategy of the organisation and partners, equity, effectiveness, greatest need, cost, deliverability, availability of evidence, delivery of outcomes tangible to the public, sustainability, transparency to the public, universal provision | 1,2,4,7,8,9,10,13,16 | Collaborative but unstructured | N (use of criteria in decision making was unstructured) | Decision makers who took part in the interviews were: chairs and Directors of 4 HWBs. They would not be the only participant in an HWB prioritisation process. | Actual (experiences of decision-makers) | | |
| **Prioritisation exercises** | | | | | | | | | | | | | | | | | | |  |
| Klamer | 2021 | Prioritisation for future surveillance, prevention and control of 98 communicable diseases in Belgium: a 2018 multi-criteria decision analysis study | To use the prioritisation method recommended by the European Centre for Disease Prevention and Control (ECDC) to rank infectious diseases, according to their relative importance for surveillance and public health, to inform future public health action in Belgium | Belgian Institute of Health (Sciensano) - the national public health institute | Belgium | Macro | Y | Prioritisation exercise | Diseases (communicable) | Specific | ECDC infectious disease prioritisation tool | Incidence, trend, case fatality ratio, severity, chronicity, absenteeism, healthcare utilisation, excess costs, public attention, spreading potential, events requiring PH action, international surveillance obligations, WHO objective for eradication, vaccine included in national vaccination programme, risk for vaccine-triggered strain replacement, existing antibiotic multidrug resistance, national reference centre/reference laboratories essential for diagnosis, congenital risks. | 1,5,8,12,14,15 | Multi-criteria decision analysis (MCDA) | Y | Epidemiologists, microbiologists, statisticians, infectious disease physicians and nurses, public health experts | Theoretical | | |
| Balabanova | 2011 | Communicable diseases prioritised for surveillance and epidemiological research: results of a standardised prioritisation procedure in Germany, 2011 | To prioritise infectious pathogens for national surveillance and epidemiological research in Germany | RKI | Germany | Macro | Y | Prioritisation exercise | Disease (Infectious pathogens) | Specific | Adapted RKI's infectious disease framework from 2004 | Incidence (illness and symptomatic infection), work and school absenteeism, healthcare utilisation, chronicity of illness or sequelae, case fatality rate, proportion of event requiring public health action, trends (incidence), public attention (Including political agenda, public perception and international duties), prevention possibilities and needs (including preventive potential and whether effective prevention strategies are well-established, with no need to modify), treatment possibilities and needs (whether medical treatment is often required and effective treatments well-established) | 1,8,11,12,13,15 | Delphi process | Y | Infectious disease experts from a range of Germany's national bodies relevant to infectious disease control, including RKI. The authors noted that future inclusion of patient representatives in the prioritisation process could expand societal perspectives. | Actual (prioritisation exercise) | | |
| Graham | 2016 | Criteria-Based Resource Allocation: A Tool to Improve Public Health Impact | To examine the appropriateness and utility of PBMA for resource allocation in a local public health setting | Middlesex-London Health Unit (MLHU) | Canada | Micro | Y | Prioritisation exercise | Programmes (proposed changes to budgets within existing programmes) | Generic | (PBMA process) | 1. legislative requirements: impact of the proposed change on the ability of the program to meet the legislative requirements for this program. 2. alignment: alignment of the proposed change with MLHU's Strategic Plan or other guidance documents. 3&4. Health need: need for the program (3 - burden of illness/risk factors, 4 - SDOH or Health inequalities). 5&6. Impact: expected impact of the proposed change on burden of disease and risk factors (5), SDOH and health inequalities (6). 7. impact: expected impact on client experience. 8. Community capacity: are others in the community doing some or all of this program/activity or is it unique to MLHU? 9. Collaboration/partnership: how does the proposed change affect collaboration/partnerships that contribute to meeting MLHU's goals? 10,11,&12. Organisational risk/benefits: assess the risks/benefits to MLHU of implementing proposed change (reputation/risk of litigation) (10), implementation challenges - ease of sustainment or impact on other frontline/support services (11), impact on workplace culture/morale/ability to innovate/collaboration (12)). | 1, 2, 7, 8, 9, 10, 13, 14 | Programme budgeting and marginal analysis (PBMA) | Y | Senior leadership team (including directors) and managers in the local public health agency and the board of health (containing citizen representatives and municipally elected councillors) governing that LPHA | Actual (prioritisation exercise) | | |
| Mitton | 2011 | Difficult decisions in times of constraint: criteria based resource allocation in the Vancouver Coastal Health Authority | To develop a plan to address a forecasted deficit in the Vancouver Communities division of the Vancouver Coastal Health Authority. | Vancouver Coastal Health Authority | Canada | Micro | Y | Prioritisation exercise (PBMA) | Not specified (areas for funding cuts) | Generic | (PBMA process) | Strategic alignment: alignment to mandate (including whether the service was only provided by and only the responsibility of VCH); efficiency (including optimal use of resources to yield maximum benefits), effectiveness and appropriateness - including whether care achieves intended outcomes and is evidence-based, impact on timely access to care for service users), flow/integration (impact on coordination with other healthcare programs to ensure continuity of care from the service user's perspective) Health impact: numbers affected by the proposed change, equity, significance of impact in terms of clinical outcomes, impact on health promotion and disease prevention, impact on client experience (including safety and effectiveness) Organisational impact: impact on workplace environment (morale, tools and equipment, teamwork, and personal and professional growth), innovation and knowledge transfer, implementation challenges, likelihood of change resulting in downstream changes in use of health care services. | 1,2,3,7,8,9,10,13,14,16 | PBMA | Y | All directors and clinical leads from Vancouver Communities (working group), broader Vancouver Communities personnel and senior executive members (advisory panel). | Actual (prioritisation exercise) | | |
| Marsh | 2012 | Prioritising investments in public health: a multi-criteria decision analysis | To demonstrate the feasibility of developing and applying a method for prioritising preventative health interventions in the UK | N/A | England | Macro and micro | Y | Prioritisation exercise | Interventions (preventive) | Generic | Framework developed in the study | Incremental cost-effectiveness (cost per QALY gained), proportion of the population eligible for the intervention, distribution of benefits (ratio of the proportion of the most disadvantaged 20% of the population eligible for the intervention to the proportion of the population as a whole eligible for the intervention), affordability (the budget required to fund the intervention if all eligible people received it), certainty (evidence quality). | 1,2,3,4,16 | MCDA | Y | Senior decision-makers (e.g. Chief executives, Directors of Commissioning, Public Health and Social Services, and leaders from other relevant national bodies) contributed to selecting the interventions to be assessed, criteria to be used and their weighting. | Theoretical | | |
| Dahl | 2015 | Communicable Diseases Prioritised According to Their Public Health Relevance, Sweden, 2013 | To prioritise infectious pathogens for resource allocation and surveillance in Sweden | The Public Health Agency of Sweden | Sweden | Macro | Y | Prioritisation exercise | Disease (Infectious pathogens) | Specific | Modified RKI framework 2011 | Incidence (illness and symptomatic infection), work and school absenteeism, healthcare utilisation, chronicity of illness or sequelae, case fatality rate, proportion of event requiring public health action, trends (incidence), public attention (Including political agenda, public perception and international duties), prevention possibilities and needs (including preventive potential and whether effective prevention strategies are well-established, with no need to modify), treatment possibilities and needs (whether medical treatment is often required and effective treatments well-established) | 1,8,11,12,13,15 | Delphi process | N (this was one modification to the RKI process) | A selection of local and national stakeholders (National Board of Health and Welfare, Public Health Agency of Sweden) and experts in communicable diseases (County Communicable Disease Control Officers) | Actual (prioritisation exercise) | | |
| Suwantika | 2021 | Multi-criteria decision analysis to prioritise the introduction of new vaccines in Indonesia by using the framework of the strategic multi-attribute ranking tool for vaccines (SMART vaccines) | To prioritise new vaccines for introduction in Indonesia | N/A | Indonesia | Macro | Y | Prioritisation exercise | Interventions (vaccines) | Specific | US Institute of Medicine and National Academy of Engineering Strategic Multi-Attribute Ranking Tool for Vaccines (SMART Vaccines 2.0) | Health attributes: premature deaths averted per year, incident cases prevented per year, QALYs and DALYs. - Economic attributes: net direct costs (savings) of vaccines per year, workforce productivity gained per year, one-time costs (development, licensure, and start-up costs), cost-effectiveness (cost per QALY gained or DALY averted). - Demographic attributes: benefit to infants, women, children, socio-economically disadvantaged, military personnel and other potentially priority populations (e.g. immunocompromised). - Public concerns: availability of alternative PH measures, potential adverse events following immunisation due to the vaccine, possibility of the disease to raise fear and stigma in the public, and serious pandemic potential. - Scientific and business attributes: likelihood for financial profitability for manufacturer, potential for demonstrating new production platforms, availability of existing or adaptable manufacture techniques, potential litigation barriers, interest from NGOs and philanthropic organisations. - Programmatic attributes: potential to improve vaccine delivery methods, fit with the existing immunisation schedule, potential to reduce challenges relating to cold chain and other requirements. - Intangible attributes: potential for eradication or elimination of the disease, potential for raising public health awareness and changing behaviour. - Policy attributes: interest for national security, potential for advancing of foreign policy goals and foreign assistance" | 1,2,3,4,7,8,9,10,11,12,13,14, 15 | MCDA | Y | International stakeholders (donors - WHO, UNICEF, GAVI), national stakeholders (ministry of health, ministry of finance, national public procurement agency, immunisation experts), and vaccine manufacturers (national and multinational) | Theoretical | | |
| **Model/framework development and case studies/evaluation** | | | | | | | | | | | | | | | | | | |  |
| Lasry | 2012 | Allocating HIV prevention funds in the United States: recommendations from an optimization model | To support national HIV prevention planning efforts and inform the decision making process for HIV resource allocation using a resource allocation model. | CDC (Division of HIV/AIDS Prevention) | United States | Macro | Y | Modelling | Programmes (HIV prevention) | Specific | CDC HIV resource allocation model | Combination of programmes giving maximal reduction in HIV incidence within the specified budget | 3 | Epidemiological modelling and budget optimization modelling | N | N/A | Actual (prioritisation exercise) | | |
| Stratil | 2020b | WICID framework version 1.0: criteria and considerations to guide evidence-informed decision-making on non-pharmacological interventions targeting COVID-19 | To develop a decision-making framework adapted to the challenges of decision-making on non-pharmacological interventions to contain the global SARS-CoV-2 pandemic | N/A | Germany | Macro | Y | Prioritisation case study (descriptive) | Interventions (non-pharmaceutical covid interventions) | Specific | WICID 1.0 framework (adapted from WHO-INTEGRATE) | (1) Implications (of the intervention or measures) for the course of the pandemic and its impact on health; (2) implications (of the intervention or measures) for quality of life, social wellbeing and mental health; (3) implications (of the intervention) for physical health, health behaviour, health risks and healthcare (eg availability, accessibility, acceptability and care quality) beyond COVID-19; (4) proportionality and implications for individual autonomy and fundamental rights; (5) acceptability of and willingness to implement the measures (among the population); (6) equity, equality and fair distribution of benefits and burdens; (7) societal and environmental implications and considerations; (8) implications for the economy as a whole; (9) resource implications and considerations (of the intervention eg masks) (10) feasibility implications and considerations (practical, technical and political feasibility and legal conformity); (11) interaction with and implications for the health system; quality of evidence (a meta-criterion) (more detailed criteria in sub-criteria) | 2,7,8,10,11,13,15,16 | Describes a suggested process, but is not reported as a specific approach. | N (no explicit scoring of criteria in this framework, although authors suggest that the "weight" of each criterion is considered in the deliberations) | Exact stakeholders to be involved in the prioritisation process were not described, although wide and equitable stakeholder engagement was encouraged. | Theoretical | | |
| Hauck | 2016 | Priorities for action on the social determinants of health: Empirical evidence on the strongest associations with life expectancy in 54 low-income countries, 1990-2012 | To identify a small set of the most influential social determinants of health using existing data and an empirical approach, in order to inform priorities for research and policy action. | N/A | 54 LICs | Macro | Y | Modelling | Risk factors (social determinants of health) | Generic | N/A | Association with life expectancy at birth | 1 | Modelling (extreme bound analysis) | N | N/A | Theoretical | | |
| Choi | 2019 | The Pan American Health Organisation- Adapted Hanlon method for prioritisation of health programs | To describe how PAHO adapted the Hanlon method to suit prioritisation of a wider range of health programs (disease and non-disease control programmes) and used it to implement the PAHO Strategic Plan 2014-2019 | Pan-American Health Organisation (PAHO) | Americas | Macro | Y | Prioritisation case study (descriptive) | Programmes (disease and non-disease (eg health system) oriented programmes) | Generic | PAHO-adapted Hanlon method | Size of the problem (prevalence or incidence of disease or extent of system or program deficiencies for non-disease oriented programmes), seriousness of the problem (urgency - trend in problem over past 5 years; severity of consequences - premature mortality or disability, loss of QoL, burden to health services; economic loss - direct and indirect costs associated with the problem; negative impact on others - people or countries - including the consequences of inaction), effectiveness of intervention (availability of cost-effective interventions, efficacy, reach/coverage, or qualitative judgement of effectiveness for non-disease oriented programmes), inequity (unjust difference in disease occurrence or access to health programmes by gender, ethnicity, income, literacy, urban/rural location, and other equity stratifiers), institutional positioning (extent to which the organisation/institution (in this case PAHO) is uniquely positioned to assist with a program need, taking into account a country's capacity and contributions of other partners; also political, strategic and technical considerations) | 1,2,3,5,6,7,9,11,12,14,15 | Adapted Hanlon method | N | Representatives from 12 PAHO member states took part in the adaptation and testing of the method. For national prioritisation sessions national government agency experts with broad knowledge of health and public health take part. | Actual (prioritisation exercise) | | |
| Longfield | 2013 | Putting health metrics into practice: using the disability-adjusted life year for strategic decision making | To describe the advantages and constraints of using DALYs averted to compare the impact of health programmes, and to describe how the approach influenced organisational strategy in an international NGO (Population Services International, PSI). | PSI | International | Macro | Y | Evaluation research | Interventions | Generic | PSI DALYs averted model | Baseline DALYs, DALYs averted | 1,7 | Modelling | N | Senior managers at global, regional and country levels of the organisation. | Actual (prioritisation/ evaluation exercise) | | |
| Simoes | 2006 | Prioritisation MICA: a Web-based application to prioritise public health resources | To describe a public health priority setting model, the Missouri Information for Community Assessment Priority Setting Model (Priority MICA), which uses epidemiologic measures available in most surveillance systems across the United States. | Missouri Department of Health and Senior Services | USA | Micro and meso | Y | Prioritisation case study (descriptive) | Diseases and risk factors | Generic | Priority MICA | Size/magnitude of the problem (the number of emergency department (ED) visits, hospitalizations, and deaths for a disease, or number of people with a given risk factor), severity (number of deaths of people younger than 65, hospital days of care, days lived with disability), urgency (trend in mortality for diseases or in incidence or prevalence for risk factors), preventability (evidence-based effectiveness score for community-based prevention interventions), community support (perceived level of community support for preventive action), and racial-disparity (ratio of black to white age-adjusted death rate and ED visit rate ratio). | 1,2,8,13 | Priority MICA model | N (not in the case study provided, but weighting is possible in the model) | Proposed users of the model include: makers of health policy and planners, such as officials at state and local public health organisations and private foundations funding public health work | Actual (prioritisation tool) | | |
| Maciosek | 2017 | Updated Priorities Among Effective Clinical Preventive Services | To identify priorities for improving delivery rates of 28 evidence-based clinical preventive services based on comparable estimates of relative health impact and cost effectiveness. | National Commission on Prevention Priorities (NCPP) & HealthPartners Institute (the research arm of HealthPartners, the largest consumer-governed, nonprofit health care organisation in the US) | USA | Macro | Y | Prioritisation exercise | Programmes/ interventions | Generic | National Commission on Prevention Priorities (NCPP) framework | **Health impact:** clinically preventable burden (total possible QALYs gained); cost effectiveness (cost per QALY gained) | 3,7 | Modelling and/or literature review to calculate the criteria values, followed by scoring based on rank for these measures | N (but authors note that they could be) | The Health Partners Institute conducted the analysis and the NCPP (consisting of clinicians, health insurance plan leaders, employers, government representatives and academics) guided the methods. The results are intended to inform decision making by e.g. clinicians, medical groups and health systems | Actual (prioritisation exercise). The authors note that clinicians and health care organisations incorporate these rankings in their quality improvement efforts. | | |
| **Non-empirical articles** | | | | | | | | | | | | | | | | | | |  |
| ECDC | 2017 | ECDC tool for the prioritisation of infectious disease threats | To enable relative ranking of different infectious disease threats using an Excel-based tool, as a supplement to support decision-making in preparedness planning. | ECDC | European Union (for the example exercise) | Macro | N | Prioritisation tool | Diseases (infectious) | Specific | ECDC Excel-based tool | Criteria can be selected by the user, but the criteria used by the ECDC in their EU risk ranking exercise were: probability of introduction of a pathogen with the potential for onward transmission in humans into the study jurisdiction in the next 5 years, peak annual estimated incidence in the study population over the next 5 years (includes consideration of available public health prevention measures), case fatality proportion at peak incidence levels (includes availability of medical intervention), probability that the risk increases in the next 5 years in the study jurisdiction, discomfort of a disease episode at the individual level, economic impact of the disease from a societal perspective (healthcare system costs, productivity loss, tourism and trade losses) | 1,5,13,15 | MCDA | Y | Multidisciplinary experts selected to reflect the objectives and scope of the prioritisation exercise (e.g. geographical and disease scope) | Actual (prioritisation tool) | | |
| Gericke | 2005 | Intervention complexity—a conceptual framework to inform priority-setting in health | To propose a conceptual framework for the analysis of the feasibility of interventions according to their degree of technical complexity to inform priority-setting in health. | N/A | Resource-poor settings | Macro | N | Concept paper | Interventions | Generic | Paper describes a new framework | Intervention characteristics: stability of the intervention product (eg vaccine), standard disability, safety profile ease of storage and transport, need for regular supplies, number and type of supplies needed, high-technology equipment and infrastructure needed, number of different types of equipment needed, maintenance needed, ease of acquiring the materials, supplies and equipment Delivery characteristics: whether services could be provided through the retail sector rather than public or private health facilities, level of hospital facility required, skill level required for service provision, skill level required, staff supervision required, frequency or duration of professional services required, management and planning requirements, dependence of delivery on communication and transport infrastructure Government capacity requirements: need for regulatory and legislative capacity, management systems and dependence on collaborative action (eg between government sectors, with civil society, or external funding agencies). Usage characteristics: need for consumer information/education to apply the intervention effectively, pre-existing demand for the intervention, risk associated with resale/counterfeiting. | 7, 8, 10, 12, 13, 14 | Not described | N | N/A | Theoretical | | |
| The Health Foundation | No Date | STAR: Socio-technical allocation of resources: guidance document | To support commissioners’ budget prioritisation processes by combining a technical value-for-money analysis with extensive stakeholder engagement and discussion. | The Health Foundation | UK | Micro | N | Prioritisation tool | Interventions | Generic | STAR (Socio-technical allocation of resources) | Description of typical patient population, number of people who will benefit from the intervention, implications of no intervention, clinical effectiveness for the 'typical patient', potential obstacles to implementation (eg internal issues such as recruitment, culture, technology or funding, or external issues such as patient uptake, political considerations), likelihood of successful implementation, cost of the intervention, health benefits of the intervention relative to other interventions, effect on health inequalities, value for money (one STAR model output) | 1,2,3,4,7,8,9,11,13 | MCDA | N | The STAR process includes a step to identify stakeholders to participate. These can include: internal stakeholders (local healthcare commissioners) and external stakeholders (e.g. patients, carers, or patient representatives, physicians, providers, community staff and managers, commissioning managers, QIPP lead, bodies such as NHS England, and other local organisations such as the local authority, charities etc) | Theoretical | | |
| Michaelis | 2002 | Priority-setting ethics in public health | To utilise the principles underlying allocation decisions in the field of organ transplantation to explore the possibility of a similar set of standards designed to guide and to explain public health funding allocation decisions. | N/A | USA | Not specified | N | Narrative review/editorial | Programmes | Generic | Organ transplantation prioritisation principles | Likelihood of benefit from the programme (does the programme use approaches acceptable to the target population, success of similar initiatives, barriers to programme success eg social or cultural), impact of programme on quality of life (including consideration of benefit for socially disadvantaged groups), available alternatives (working with the community to ensure that the community's own resources can be used to alleviate existing public health problems), duration of the benefit of the programme, number of beneficiaries from the programme, urgency of the patient/population's need (number of people affected by the problem and the severity of its medical/social consequences) | 1,2,7,8,14 | Not described | N | Public health professionals (not further specified) | Theoretical | | |
| Mock | 2010 | Developing priorities for addressing surgical conditions globally: furthering the link between surgery and public health policy | To present preliminary ideas on how to better define priorities for surgical care, in particular for conditions which interface with public health. | N/A | International | Macro | N | Concept paper | Interventions (surgical) | Specific | Describes preliminary ideas for a framework | Preliminary criteria: public health burden, effectiveness of the surgical procedure, cost-effectiveness of procedure including related ancillary services and treatments, feasibility of promoting the procedure globally including in resource-constrained environments (based on complexity of the procedure, training required to perform the procedure, need for costly equipment, likelihood the procedure can be performed safely with a low complication rate, need for ancillary services and additional treatments) | 1,3,4,7,13 | Not described | N | N/A | Theoretical | | |
| Neiger | 2011 | Basic priority rating model 2.0: current applications for priority setting in health promotion practice | To describe the latest published version of the BPR model (also known as the Hanlon model) and suggest revisions to this model to improve it (e.g. its applicability to chronic as well as communicable diseases, and to use data currently available to decision makers) | N/A | USA (the example provided) | Macro (the example provided) | N | Prioritisation case study (descriptive) | Diseases (in the example provided) | Generic | Adapted Hanlon method | Size of problem (prevalence, incidence, or mortality), seriousness of problem (urgency - trend in mortality; severity - lethality, premature mortality, disability; economic loss - societal costs including spend on the disease; impact on others- eg communicable nature of disease, behavioural impacts such as secondhand smoke, or impact on caregivers); effectiveness of interventions; PEARL (proprietary - does it fall within the organisation's scope, economics, acceptability, availability of financial resources needed, and legality) | 1,4,5,7,8,9,10,15 | BPR 2.0 (adapted Hanlon method) | N | Not described | Theoretical | | |
| Ogilvie | 2011 | Assessing the Evaluability of Complex Public Health Interventions: Five Questions for Researchers, Funders, and Policymakers | To present a set of questions to stimulate and structure debate among researchers, funders, and policymakers and help make decisions about evaluation within and between complex public health interventions as they evolve from initial concept to dissemination of full-scale intervention packages | N/A | UK | Macro | N | Concept paper | Interventions (complex) | Generic | Paper describes a new framework | **Where is a particular intervention situated in the evolutionary flowchart of an overall intervention program?** [classified as: concept/idea/proposal, in development, concrete developed intervention, implementation of multiple instances of the same principle, and roll-out] (Referencing the socioeconomic/sociopolitical context; the degree to which it is embedded in organisational contexts; how early it is in its development as interventions shouldn’t be evaluated too early in their development as they haven’t garnered enough evidence of effectiveness etc); **How will an evaluative study of this intervention affect policy decisions?** (If the outcomes of the evaluation have no 'customer' to use them/effect on policy decisions, then there is no use conducting them; inter-sectoral engagement is needed where programmes non-health impacts; the potential of the evaluation to be of wider public interest even if it seems "blue sky" also needs to be considered; consideration of whether the new data required to alter policy relates to the acceptability, implementation, reach, uptake , mechanism or dissemination of an intervention, or to a cost-effectiveness/cost- benefit analysis); **What are the plausible sizes and distribution of the intervention's hypothesised impacts?** (the interventions most worth evaluating are those expected to have a large effect on a large number of people; small scale, novel or untested interventions should still be evaluated if they e.g. could have important adverse effects, benefits in non-health areas, effects which may contribute to widening or narrowing health inequalities, are scalable to widespread implementation, or a promising new type of intervention); **How will the findings of an evaluative study add value to the existing scientific evidence?** (No need for evaluation if the intervention is already well/widely researched unless evidence is lacking for its effects on the population in question, different outcomes including non-health outcomes, the mechanism for its effects, scalability, sustainability, generalisability or distributional effects ie impact on inequality); **Is it practicable to evaluate the intervention in the time available?** (Is there enough time and resource to carry out an evaluation, is it feasible to collect data for evaluation, has the intervention been around long enough, how long will it take to get the results and will they still be useful by then, will it be possible to isolate the intervention's impact from other factors, has the context or content of the intervention changed in a way that would allow useful comparisons?) | 2,3,4,7,8,9,11,13,14,16 | Not described | N | The exact stakeholders who should be involved in the prioritisation process were not specified, but the authors report that the decision-makers who might consider these criteria include researchers, funders and policy makers. They also note the need for cross-sectoral engagement when evaluating programmes that have non-health impacts/components (eg education, transport). | Theoretical | | |
| Public Health England | 2018 | The prioritisation framework: making the most of your budget | To help local authorities conduct a systematic prioritisation exercise and get the best value for money. | Public Health England | England | Micro | N | Prioritisation tool | Programmes | Generic | PHE Prioritisation Framework | Routine criteria are: current state of programmes (current investment in the programme area compared to other local authorities, current outcomes in the programme area relative to other local authorities), feasibility of reaching the programme's potential (current context, requirement for budget shift, impact of budget shift on outcomes). Most criteria relating to value for money in the future are not pre-specified, but example criteria given include: effectiveness, cost-effectiveness (achieving outcomes for lower cost than alternatives), impact on inequalities, local need (e.g. how many people the programme benefits), impact on prevention, legal requirements, acceptability (political, cultural, moral), innovation, building community assets to support health and wellbeing, impact on local economic growth, impact on other parts of the health system, wider impacts (e.g. on crime), rigour and certainty of evidence. | 1,2,3,4,7,8,9,10,11,14,15,16 | MCDA | Y | Local public health stakeholders including local public health teams | Actual (prioritisation tool) | | |
| Rasul | 2020 | A Framework for Improving Policy Priorities in Managing COVID-19 Challenges in Developing Countries | To present a framework for identifying and prioritising policy actions to address the COVID-19 challenges and ensure sustainable recovery in developing countries | N/A | Developing countries | Macro | N | Concept paper | Policies | Specific | Describes a new framework | Saving human lives and livelihoods (reducing health risks, improving health care, reducing communicable diseases, and ensuring provision of basic health services and human needs), efficiency and effectiveness (cost effectiveness, maximising health and social benefits), equity and fairness (protecting those most at risk and serving the most deprived), sustainability and resilience (long-term social, economic, and environmental benefits, building capacity to deal with future challenges), synergies and trade-offs with other strategic objectives (e.g. increasing employment and income), aligning policy instruments (coherence, compatibility, congruence; reconciling private and societal good) | 2,3,7,8,15 | Stakeholder discussion and agreement using "principled engagement" | N (but suggested they could be weighted based on country context) | Government actors including key government agencies including health, finance, security, water, food, trade and commerce; experts and development partners, think tanks, and non-government actors | Theoretical | | |
| Wani | 2020 | Key steps in planning and evaluating an effective program for public health | To present an outline for planning and evaluating an effective public health program | N/A | Not specified | Not specified | N | Narrative review/editorial | Programmes | Generic | Presents its own framework | Importance of the condition being addressed: mortality, morbidity, distress, treatment costs, economic loss; intervention success; intervention costs | 1,4,5,7,15 | Simple scoring system | N | Not explicitly reported, although it was noted that community leaders, peers and volunteer groups should be involved in the planning process. | Theoretical | | |

**Supplementary table 2: Data extraction table for papers addressing the use and utility of prioritisation tools and frameworks**

| **Author** | **Year** | **Method for evaluating use/utility (outcome)** | **Outcomes evaluated/ mentioned** | **Summary of findings relevant to RQ3** |
| --- | --- | --- | --- | --- |
| Hunter | 2019 | Qualitative (interviews, observation, and thematic analysis) | Stakeholder perception of utility | **Stakeholder perception of utility:** The PF process was used by all 3 LAs to inform budget allocation, most recommendations “seemed to be based on evidence and the scoring systems inherent in the PF". The alignment between PF framework scores and final budget decisions in one LA in particular led study authors to conclude that "the use of the PF and the associated evidence and scoring has been a key driver in terms of the rationale for budgetary changes". The tool was reported to be welcomed by the LAs which adopted it, with users considering it a useful tool. Its systematic framework, collaborative and transparent approach were seen as beneficial. However, users noted that the process was time consuming, and that the political context for making these decisions (i.e. local government) could hinder the adoption of the tool, as elected officials make the final decisions. **Use:** concluded that "the tool proved itself to be sufficiently robust to be adopted more widely by local authorities and their public health teams." |
| Gilsdorf | 2011 | Survey | Stakeholder perception of utility | **Stakeholder perception of utility**: Most participants in the survey considered prioritising pathogens useful for public health purposes (68/72), for surveillance and epidemiological research (64/72), clinical research (57/72). Most also considered prioritisation to be beneficial for: public health services at a national level (58/72), international level (49/72). Some thought it would be beneficial to: regional public health services, universities and ministries of health, to guide surveillance and research agendas (33/72), and local public health services (29/72). Most participants deemed all criteria useful. The definitions of some criteria were considered unclear for scoring purposes. |
| Mitton | 2011 | Interviews with decision makers | Ability to provide recommendations for disinvestment to address budget deficit; stakeholder perception of utility | **Utility for budgeting:** The exercise was able to generate recommendations for 44 disinvestment initiatives with an annual value of $4.9 million Canadian dollars, all of which were agreed by the Senior Executive. **Stakeholder perception of utility**: All respondents were reported to be extremely positive about framework implementation and resulting recommendations. They also felt that it would have value in developing investment and re-allocation plans, and not just disinvestment plans to balance the budget. The interviewees felt that wider rollout in the health authority should happen because of its effectiveness in the pilot, and it would lead to more consistency in resource management and opportunities for sharing knowledge across the organisation. |
| Graham | 2016 | Focus group & survey; Qualitative (trust) | Stakeholder perception of utility | **Stakeholder perceptions of utility**: Staff trust was assessed. It was felt that transparency of the process improved trust between the board and the senior team, as did the positive local media coverage of MLHU's 2014 budget process. Some managers reported this trust may have been gained at the expense of staff trust. Staff were pleased with the improved impact of MLHU resources, but those whose programs were proposed for disinvestment reported concern and disapproval. PBMA was found to be a useful tool (as part of a broader strategy) for improving impact and transparency of resource allocation decision making. |
| Marks | 2013 | Interviews and survey | Use, stakeholder perception of utility | **Use:** At the point of this study (2008-2010), there was no formal process for prioritising investments in PH in England. Commissioners were using a variety of different approaches and decision-support tools (including locally developed tools) to generate and compare business cases for prioritisation of investments. The authors concluded that their study's results highlighted the limitations to existing tools for prioritising PH investment, skills shortages among commissioners (eg PBMA was reported to be difficult to do, weighting of criteria difficult to agree), and lack of real-time data to inform decisions. **Utility:** Commissioners considered the wider decision-making context to be more important than specific tools and techniques. The review of tools suggested that they should be critically assessed for their relevance to important principles in investment in health such as equity, longer-term health gain, SDOH, and intersectoral collaboration. A combination of tools may be required to ensure that all of these aspects are taken into consideration. |
| Otim | 2014 | Interviews and survey | Stakeholder views of challenges, author opinion on use | **Stakeholder views of challenges**: stakeholders pushed for more community engagement to enhance acceptability of results. They also reported that their priority setting process/framework is not performing well, as it needs a more systematic, transparent process, there is also a need for a more timely and strong evidence base, upskilling of staff, and mechanisms for shifting resources. The study revealed that criteria identified by participants were similar for the two organisations; however, there were differences around the place for equity. The differences are likely to stem from the different primary roles that the two organisations are engaged in. **Author opinion of use**: The results from this study are likely to inform the debate on the appropriateness of the existing criteria for priority setting, not only in Australia but other countries, given the scarcity of the resources. |
| Jimenez-Soto | 2012 | Systematic review and recommendations based on findings | Evidence on utility | **Evidence on utility:** The authors noted that "even in high-income settings where participatory, accountable and rational approaches to priority-setting in healthcare are achievable, the process and outcomes of such exercises have been unsatisfactory." They state that very few approaches for evidence-based priority setting have been evaluated in either high-income or LMIC settings, and that there was lack of guidance on how best to do such evaluations. They identified three frameworks for evaluating priority setting |
| Leider | 2014 | Semi-structured interviews and survey | Stakeholder reported use | **Use:** There was variability in prioritisation/resource allocation processes, with only 29% of respondents in the survey saying that they used prioritisation or decision tools as part of the process very often or often in the past year. Use was more common among executives than division directors (any use: 86% vs 74% respectively, p=0.049). In interviews only one out of the 6 SHAs was reported to systematically use a priority-setting framework. Those who did use it reported favourable impressions of it, and used it to provide additional perspectives rather than solely relying on its results to prioritise programmes. The other SHAs either did not use such tools (2 SHAs), or only some respondents reported using such tools (3 SHAs). |
| Balabanova | 2011 | Anecdotal | Author perception of utility and potential uses, impact on decision making | **Author perception of utility:** The authors reported that the prioritisation process "worked well", and that its modular structure could make it useful for other settings. They felt that the process allowed them to conduct prioritisation independent of programmatic views and compare pathogens both within and across different disease groups. The comprehensive and transparent approach meant that the results are defensible. **Utility:** The list of ranked pathogens established here were intended to inform mid-term strategic decisions, including strengthening the existing or introduction of new surveillance systems for pathogens from the high priority group and re-consideration of the research and surveillance needs for those from the lowest priority group. It was reported to have influenced the decision process on the need for the installation of new and continuation of existing national reference centres in Germany and the internal planning for the respective allocation of resources. **Author perception of use:** The prioritisation tool or its components can be applied across different areas of infectious diseases and different geographical areas |
| Dahl | 2015 | Comparison to existing surveillance efforts in Sweden | Validity compared to current resource allocation, author perception of utility | **Utility:** The authors found that 95% of the FTEs for pathogen surveillance through notifications were spent on the pathogens in the highest and the high priority groups, suggesting that the PHA was already focused on the most important pathogens. However there were some (two) pathogens in the highest priority group which did not have any surveillance through notification while some (eight) in the low priority group did. **Author perceptions of utility:** Results of the prioritisation "should mainly function as an indication that the need for surveillance, or lack of surveillance, for certain pathogens should be evaluated" and note that the PHA would be doing this. |
| Kapiriri | 2022 | Review of national covid-19 plans | Use, author perception of utility | **Use:** All of the countries' plans contained information on some aspects of the expected priority setting parameters within the 5 framework domains (priority setting context, prerequisites, priority setting process, implementation, and outcomes/impact) but none of them addressed all 20 parameters in these domains. None of the 18 countries used clear priority setting processes or tools (one of the quality parameters), although several reported following the WHO recommended strategy for pandemic planning, which itself does not include priority setting. Less than half (7/18) of the countries' plans included explicit priority setting criteria (another of the quality parameters). The study did not explicitly compare quality of the priority setting process and outcomes of the covid-19 pandemic. **Author perception of utility**: The authors recommended that countries should consider priority setting as a critical part of their routine health emergency and disease outbreak plans, ensuring that it is integral to pandemic planning, response and recovery. |
| Longfield | 2013 | Anecdotal | Use in informing strategic decisions and monitoring | **Use**: Senior managers at all levels of the organisation- global, regional, and country- use these results to monitor progress against intended targets and to guide strategic decision making. They identify areas for improvement and redirect program and funding priorities as needed. The DALYs averted measure was factored into individual performance goals, annual appraisals, and incentive compensation, in addition to the country and regional operating plans. |
| Simoes | 2006 | Anecdotal | Author perception of potential use | **Author reported potential use:** Priority MICA allows all public health agencies, local and state, and their communities to prioritise resources because the data are readily available through existing surveillance systems. |
| Stratil | 2020(a) | Anecdotal | Author perception of potential use | **Author reported potential use:** The authors reported that to the best of their knowledge, this was the most comprehensive and up-to-date list of real-world criteria available for health decision-making. It could therefore provide a valuable tool for informing decision-makers wishing to select those criteria relevant for a given type of decision and decision-making context. This comprehensive list is likely to be most relevant to decisions in public health or healthcare. Due to the focus of the present study, the applicability for research priority setting or the evaluation of diagnostic or testing devices is likely to be more limited, as it may not have covered all relevant publications. It can serve as a resource when considering which criteria to include in sound multi-criteria approaches and how to use these. |
| Choi | 2019 | Validation against an alternative approach (Delphi) but details of methods and results were not provided. Reporting of implementation in the region. | Author perception of potential use, validity in comparison to other methods | The PAHO-adapted Hanlon method was approved by the PAHO Directing Council for implementation across the Region. **Author perception of use**: The adapted method is more relevant and useful to the wider scope of health and public health. The method may also be applicable to WHO, other WHO Regions, country governments, and other health institutions, all of which face similar needs in prioritising both disease- and non-disease-oriented programs. **Validity against another method:** The PAHO adapted method was reported to have been validated against the ranking results from a more time consuming Delphi exercise carried out in pilot testing with a group of senior managers. |
| Wanjau | 2020 | Stakeholder workshop | Stakeholder identified challenges | **Stakeholder identified challenges**: the stakeholders identified political bias, financial constraints, reactionary priority setting, external influences (the pharma industry), and religious and cultural conflicts as a perceived challenge in the priority setting process (prioritising health issues that affect prominent people in society). |
| Greaves | 2017 | Paired interviews | Use | **Use:** Criteria were not explicitly referenced in the decision making of the boards which instead made unstructured prioritisation of population sub-groups or interventions agreed by consensus. While there were some key similarities between HWBs in the criteria they used, there were also clear differences, and the authors noted that it was unclear how rigorous or systematic these approaches were. |
| Kaur | 2019 | Anecdotal | Author perception of potential use | **Author reported potential use:** The authors suggested that process criteria should be incorporated to aid decision making. They also suggested that in countries with decentralised health systems, macro level priority setting should be thought about in terms of implementation at meso and micro level, and that there should be more involvement of patient organisations, civil society (public and community stakeholders) to justify and inform decisions. |
| Stratil | 2020(b) | Anecdotal | Author perception of potential use | **Author perception of potential use**: The WICID framework is intended to be applicable across a broad range of NPIs and decision-making contexts, and the authors suggest that it can be used as a ready-to-use framework, or as a guide to systematically reflect on NPIs and their interdependencies, and adapt them based on the specific needs of the decision making process. The authors provide some brief guidance on how this could be applied. The WICID framework was designed to align with other public health ethics frameworks (it translates values and public health ethics framework principles into criteria). |
| Platonova | 2010 | Survey | Utility (congruence between method and stakeholder view of criteria importance) | **Utility:** Agreement on the rank of importance of the criteria varied due to magnitude, seriousness and political pressure. 22 participants (63%), there was congruence between how priorities were actually set in their departments and what criteria they considered important. However, for 13 participants (37%), there was a difference between what was done in their departments and what criteria they thought were important. It was determined that 11 officers (31%) predominantly used subjective criteria in real practice but considered objective criteria as more important for priority setting. |
| Zhao | 2022 | Publication metrics and analysis | Use, utility in informing decision making | **Use:** The MCDA studies showed a relatively consistent rate of publication between 2010 and 2021. The articles mainly came from Canada (20%, 11 studies), multiple EU countries (9%, 5 studies), UK (7%, 4 studies) and Turkey (7%, 4 studies). (NB Only studies from OECD countries were eligible for inclusion in the review). Often studies highlighted that their tool/framework could be re-used over time, and were applicable in different geographies or contexts. Most studies used prioritisation to inform decisions about disease management, such as resource allocation (61%, 33 studies), and most focused on the topic of infectious diseases (61%, 33 studies). Most studies focused on the development and (typically pilot) testing of frameworks/tools was the focus on 57% of studies (31 studies), whereas in 43% (23 studies) the aim was to inform health-related policies and practices. **Utility:** Almost half of the studies (46%, 25 studies) reported that MCDA was "beneficial to the decision-making process", and in others which did not explicitly report usefulness often the benefits listed outweighed the limitations as reported by the authors. The benefits highlighted included being a systematic, transparent, and flexible way to support decision making, and offering an "effective focal point for collaboration [between stakeholders] by fostering evidence-based and structured multi-disciplinary deliberation and knowledge exchange". |
| Lasry | 2012 | Anecdotal (qualitative & quantitative) | Author reported utility and use | **Author reported utility and use**: The resource allocation model is intended to improve the allocation of funds. The results have, in part, provided the impetus for other programs, such as the Expanded HIV Testing Initiative, which has achieved a return of $1.95 for every dollar invested by CDC. |
| Ogilvie | 2011 | Anecdotal | Author perception of potential use | **Author perception of potential use:** The authors intended for this framework of questions to be useful among researchers, funders, and policymakers and to better help them make decisions about whether to evaluate certain public health interventions. They suggested that it could be used to identify what types of knowledge can be generated from evaluation and how these can support more systematic resource allocation decisions, and to provide structure and stimulate debate by all relevant parties. |

**4 Domains and their interpretation**

The table below summarises the 16 domains into which criteria were categorised. Notes on how these domains were interpreted are given in the central column. In the final column the criteria which were reported in the individual studies are listed against the domain into which they were grouped. Similar or identical criteria have been merged.

**Supplementary table 3: Domains and their interpretation**

| **Domain** | **Domain definition/notes on interpretation** | **Criteria classified within this domain from included studies** |
| --- | --- | --- |
| Burden of disease | Includes any measure of number of people affected and/or severity of disease, whether it is life threatening, pandemic potential, infectiousness etc. Includes measures of health service need/demand/use that are not explicitly translated into health system costs e.g. risk of admission to hospital, need for healthcare. **Does not include the economic burden of a disease, which is captured in other domains.** | Incidence  Prevalence  Trends in indicators of burden over time  Affects population level health (e.g. environmental health or emergency preparedness)  Disease impact on priority populations (infants, children, military personnel, or other)  Population density in an affected region  Geographically vulnerable regions  Preventable illness and disability over time  DALYs caused by the disease  Days lived with disability  Discomfort of the disease/distress caused at an individual level  Disease severity  Chronicity of the disease or its sequelae  Case fatality rate/ratio  Number of deaths/life years lost from the disease  Premature mortality/impact on life expectancy  Disease requires medical treatment or preventive action Untreated prognosis  Urgency of the problem  Population vulnerability  Pathogenicity  Transmission dynamics  Spreading potential/transmissibility/infectiousness  Evolutionary potential  Proportion of disease events requiring public health action  Risk for vaccine-triggered strain replacement  Congenital risks  Pandemic/outbreak potential  Probability of pathogen introduction  Probability of risk increasing  Capacity to detect the threat  National laboratory essential for diagnosis  Population/community need, including size of population affected and current outcomes being achieved in a programme area  Emergent issue or potential for emergence  Data from surveillance and research  Health care utilisation (e.g. ED visits, hospitalisation)/severity of health and health system consequences/predicted illness and service use  Impact of disease/risk factor on people without the disease/risk factor  Proportion of the population eligible for the intervention being assessed  Emergence of antimicrobial resistance  Benchmarking of the problem or in key performance indicators to similar areas  Numbers affected by a proposed change in services |
| Equity/Fairness/ Ethics/ Equality | Includes considering any issues relating to equity, fairness, ethics or equality. This could relate to characteristics such as gender, sexual orientation, ethnicity, education or wealth, or to reducing inequalities such as poverty reduction.    Includes both issues relating to the disease/risk factor itself (i.e. whether they disproportionately impact vulnerable groups), and issues relating to the intervention (e.g. whether it specifically targets and reduces inequity). | Disease impact on socioeconomically disadvantaged or women  Health needs by economic group, ethnic group and area  Gaps in local vs national life expectancy  Disease affects a targeted/vulnerable patient group  Racial disparity in death rate and ED visits  Unjust inequity in disease occurrence or access to health programmes  Interventions needed to narrow gaps  Social determinants of health (SDOH) and inequities addressed by the programme  Impact of programme on SDOH  Impact on inequalities in e.g. health, wealth (i.e. poverty reduction)  Equity promoting programme/intervention  QoL benefit for disadvantaged groups  Distribution of benefits by disadvantage  Meeting diverse population need  Ethical considerations  Equity (e.g. social equity, equity in access)  Equality  Fairness e.g. fair distribution of benefits and burdens  Universal/inclusive provision  Gender participation in decision-making  Influence on decision-making of ethnicity/education/wealth  Possible impact of intervention on inequalities/distribution of impacts |
| Cost-effectiveness/value for money | Includes criteria explicitly considering/calculating the balance between costs and benefit of an intervention/programme e.g. using a model, formula, graph to calculate or show incremental cost effectiveness ratios (ICERs), cost per quality-adjusted life year (QALY), or cost per other benefit etc. This included criteria reported in studies simply as “value for money” or “cost effectiveness” with no further explanation. | Optimal use of resources to yield maximum benefits  Cost per QALY gained and DALY averted  Cost-effectiveness  Cost-utility  Impact of investment on health outcomes and service use  Return on investment  Value for money  Impact of budget shift on outcomes  Availability of cost-effective interventions  Incremental cost-effectiveness ratio (ICER)  Cost-effectiveness/cost-benefit data may be needed to influence policy  PBMA analysis |
| Budget impact of the intervention/ programme | Includes the budget impact of investment or disinvestment in the programme for the body funding it. | Budget impact  Savings with intervention use  Costs of intervention/programme - total, per capita, recurrent and capital/one-off costs (e.g. costly equipment)  Opportunity cost  Balance of investment across treatment and prevention)  Affordability  Current budget allocation to a programme area  Size of budget request relative to previous allocation  Need for budget shift  Availability of funds  Financial sustainability  Ability to maximise/leverage funding for other programmes  Financial context (appropriateness)  Budget impact if all eligible people receive the intervention Economic impact of control measures for the disease (i.e. wider economic impact)  Resources available for the evaluation |
| Cost of disease from a health system perspective | Specifically, the health system cost of providing care for people with the disease - i.e. economic burden of disease for the health system. | Economic burden of risk factors on health services  Healthcare system/care/treatment costs relating to the disease  Economic impact the disease  Cost/spend/economic impact of the condition/disease  Direct costs of the disease |
| Cost of disease from patient perspective | Specifically, the cost of obtaining care from the patient perspective. | Out of pocket expenses  Risk of catastrophic costs  Cost and financial impact on beneficiaries  Direct costs of problem for patients  Affordability from a patient perspective |
| Health and wellbeing impacts of intervention/ programme | Includes both positive and negative health outcomes of the intervention/programme  E.g. effectiveness/efficacy of intervention/programme, including comparative (dis)advantage versus other options, side effects or harms associated with the intervention/programme.    Also includes changes in e.g. knowledge and behaviour that have the potential to lead to changes in health outcomes. | Balance of health-related benefits and harms  Effectiveness/efficacy  Significance of impact on clinical outcomes  Impact on burden of illness (e.g. incidence, premature deaths, DALYs, pathogenicity, course of a pandemic, QoL, wellbeing, mental health, physical health, health behaviour, health risks) or risk factor addressed QALYs gained  Life years saved  Safety (e.g. vaccine associated complications, complication rate of a procedure)  Potential for disease elimination or eradication  Potential to raise public awareness and improve behaviour Health impact including preventive impact/potential, impact on health promotion  Immediate benefit  Number of people who will benefit from the programme/intervention  Duration of benefit from the programme  Success of similar initiatives  Impact on health risks, health services, communicable diseases and basic human needs  Reach/coverage of the intervention or programme  Size of the hypothesised impact, including number of people impacted and effect size, intervention reach/dissemination, potential for adverse effects |
| Social aspects | Acceptability of the intervention/programme to the community; fear/risk perception/stigma of the disease itself, or public perception/interest in the disease/issue, which would suggest interest in/acceptability of (or interest in) measures to prevent or treat it. Impact of a programme/intervention on wider societal outcomes.    Also includes community-centeredness of programmes including building community capacity. | Public attention/perception/expectations/scrutiny/awareness/concern/ views/interests  Fear and stigma of the disease targeted  Direct public involvement (e.g. in needs assessment)  Acceptability/support/engagement of the community for the programme/intervention (including social, cultural and moral acceptability)  Community-centeredness and community strengthening of the programme (e.g. building community assets to support health and wellbeing)  Impact on locals  People's culture, background and religion  Reflection of public values  Acceptability by beneficiaries  Impact on service user (patient) experience  Patient uptake rates  Any cultural or social barriers affecting likelihood of programme success  Wider societal/intersectoral impacts e.g. on crime  Wider benefits of the intervention to carers and families Programme/intervention achieving wider societal outcomes e.g. school readiness, return to employment after illness Demands, interests and pressures from advocacy groups, beneficiaries and patient representatives  Societal considerations/implications  Media reaction  Experiences and needs of the prominent people in society  Influences from the general public  Delivery of outcomes tangible to the public  Wider public interests in conducting the evaluation  Non-health impacts of the intervention  Data needed on acceptability/uptake of the intervention  Transparency to the public  Willingness to implement the measures among the population  Proportionality and implications for individual autonomy  Pre-existing demand for the intervention  Need for collaboration with civil society  Societal disruption potential  Societal benefit potential  Alignment of policy instruments to achieve synergies with wider strategic objectives (e.g. increasing employment and income) Long-term social, economic, and environmental benefits  Building capacity to deal with future challenges |
| Organisational/ provider/ industry considerations including acceptability | Acceptability and impact of the intervention/programme to the public health organisation carrying out the prioritisation, providers such as healthcare professionals or wider healthcare industry bodies. Includes whether the intervention is included in clinical guidelines & practices, or if there is evidence of variation in practice, and whether a disease is difficult to manage. It also includes alignment with organisational priorities/strategy | Alignment with organisation’s strategic and financial priorities/plans/objectives  Alignment with federal or other guidance or local policy  Impact on partnerships/collaborations  Internal organisational risks to implementation e.g. impact on internal workplace environment/culture/morale/ability to innovate or transfer knowledge/collaboration/capacity Local targets  Organisational culture, history, philosophy and partnerships  Programme critical to the organisation's PH mission  Impact on personnel and partners; alignment with strategic plan; Special interest groups involved  Impact on other parts of the health system  Acceptability to providers  Demand, interests and pressures from those providing the intervention and by industry  Considerations of management and organisation of health system Strategic considerations  Technical considerations  Impact on organisational technical expertise/capacity  MoH policies and plans  Industry focus on treatment  Stakeholder engagement/views/influence (including of technical experts, staff, colleagues in other departments, health professionals providers, board of health, state health department)  Consistency with strategy of organisational partners  Reluctance to lay off employees  Propriety - whether the programme falls within the agency's scope  Alignment with wider Council (i.e. organisational) strategy Likelihood of resulting in downstream changes in use health care services  Impact on timely access to services  Coordination with other healthcare programmes Impact to organisational credibility/reputation  Alignment with other initiatives  Impact of climate change  Likely profitability for the manufacturer  Degree to which interventions are embedded in organisational contexts  Ability of the evaluation to impact policy |
| Legal & regulatory framework | Any legislative or regulatory requirements or issues relating to the intervention/programme which affect provision/implementation. Includes any national level decisions about inclusion of an intervention in provision (e.g. inclusion of a vaccine in the national vaccine programme) which need to be complied with at regional/local level. | Compliance/alignment with legislation (including local ordinances) Litigation risk  Mandated function/service or contributing to a statutory function  Legal feasibility/legality  Human and individual rights  Implications for fundamental rights  Legal and regulatory framework conducive for implementation  Need for regulatory and legislative capacity |
| Political considerations | Any political considerations, including alignment with government policy. | Political  considerations/context/influence/attention/interests/pressure  Political economy  Political acceptability  Political 'hot spots'  Political agenda  Political will  Political feasibility  Relevance for national security, preparedness and response  National targets  Federal priorities  Ministerial views  Input from the county council  Local policymaker and board of health political influence  Socio-political context |
| International support/donor acceptance | Alignment with international policy or donor strategy/priorities, including acceptability of prioritisation of the disease or of the intervention/programme. | WHO objective for eradication or elimination  International surveillance obligations  Interest from NGOs and philanthropic organisations  Impact on foreign policy goals and foreign assistance  International duties  Views of international development partners  Donor and global interests and pressure  Impact on other countries  Donor funding  Influence from external stakeholders such as international bodies  International support/donor acceptance  Need to collaborate with external funding agencies |
| Feasibility of implementation | Any factors affecting/relating to the feasibility of implementing an intervention/programme.    For example, this can include whether an effective prevention or treatment is available for a given disease. For an intervention/programme this could include e.g. What proportion of the affected population the programme/intervention could target? The ability to provide quality care, any capacity constraints, or any technical issues.    (Does not include any feasibility considerations falling under other domains e.g. budget impact). | Feasibility/deliverability/implementation considerations (including technical feasibility, feasibility of correcting the problem, issues relating to intervention characteristics and complexity, delivery characteristics, government capacity requirements, and usage characteristics)  Sustainability (including environmental)  Environmental considerations  Interaction with and impact on the health system  Leveraging existing or new manufacture techniques  Potential to improve delivery methods  Fit with existing immunisation schedule  Reducing cold-chain and related challenges  Demonstrating new production platforms  Possibility of reaching the potential for the programme area  Workforce and market capacity exist or can be developed  Prevention and treatment possibilities (e.g. availability/lack of suitable alternatives) and needs  Material and human resource implications  Risks to implementation including need for recruitment or technology Feasibility of promoting the procedure in resource-constrained environments (e.g. complexity of the procedure and training required) Need for ancillary services and additional treatments Limitations/lack of alternative interventions or unmet needs  Practicality/feasibility of an evaluation including: the time needed and available for the evaluation  Whether the impact of the intervention can be isolated from other factors  Stage of development of the intervention  How long the intervention has been in place  Changes in the context or context of the intervention over time  Potential scalability of the intervention |
| Current service provision | Existing provision of a given (or related) programmes/interventions/ services in the community. For example, whether the service is also being provided by other organisations in the community. Also whether the current service provision could be maintained e.g. in an emergency | Alternative service provision in the community (including through the retail sector)  Vaccine for the disease included in national vaccination programme  Availability of alternative PH measures  Existing and desired service provision  Access to services  Existing intervention coverage e.g. type and number of visits and quality of care  Service provision in the private sector  System strengthening  System benefits/interdependencies - connectedness with other programmes and services  Links with other council work  Innovation: able to improve current practice and better meet local need  Whether service can be delivered by others  Whether a programme is new or expanding versus maintaining effort  Service continuity of routine essential services  Current state of programmes  Innovativeness  Programme deficiencies  Innovativeness of the intervention |
| Societal costs of the disease | Costs to society as a whole e.g.   - Absenteeism (from work or school) - Impact on productivity - Need for informal care - Impact on wider industries and the economy | Cost to society  Impact on economy (including the local economy)  Impact on absenteeism - work and school (including for carers)  Impact on productivity loss  Impact on livelihoods (including job creation)  Impact on industries such as tourism and trade  Indirect (societal) costs |
| Evidence considerations | Issues relating to the evidence-base, such as availability/strength/quality of evidence. | Quality of evidence  Strength of evidence (e.g. rigour and certainty)  Use of evidence  Evidence considerations  Validity of epidemiologic information  Availability of research evidence and data  Existing evidence on the intervention and how evaluation would add to this |

**5 Quality appraisal**

We used the QuADS tool for appraisal of the included empirical studies.

The QuADS criteria and guidance for scoring can be found in Harrison et al. 2021. Papers which described no research component or empirical methods were not quality appraised (e.g. non-systematic reviews, editorials and opinion pieces; concept papers proposing a new or adapted/modified process or tool; guidance or descriptions of toolkits and frameworks where the information presented in the document did not present the methods and process used to develop them).

An iterative approach was used when applying the tool to each study design. The content examples provided for each criterion score in the QuADS template were used to guide appraisal, but there was still a need for some consideration of the most applicable study feature to consider within each question for each study design. We recorded specific interpretations for each question for each study design, and applied these tailored interpretations when appraising subsequent studies of the same design.

In the original QuADS tool, studies are scored from 0 to 3 (lowest to highest quality) on each questionnaire item. However, there are concerns about the application of numerical scoring to quality appraisal tools, and it is not utilised within e.g. the Cochrane risk of bias tool or the Critical Appraisal Skills Programme (CASP) tools. As such, instead of scoring we utilised a colour-coding system to indicate quality. Across all studies and designs this can be interpreted overall as the quality measure being met:

- not at all (red)
- very slightly (orange)
- moderately (yellow)
- completely (green).

The results of the quality appraisals are shown in Supplementary Table 4 below.

**Supplementary table 4: Quality appraisal results**

| **QuADS criteria** | **1. Theoretical or conceptual underpinning to the research** | **2. Statement of research aim/s** | **3.Clear description of research setting and target population** | **4. The study design is appropriate to address the stated research aim/s** | **5. Appropriate sampling to address the research aim/s** | **6. Rationale for choice of data collection tool/s** | **7. The format and content of data collection tool is appropriate to address the stated research aim/s** | **8. Description of data collection procedure** | **9. Recruitment data provided** | **10. Justification for analytic method selected** | **11. The method of analysis was appropriate to answer the research aim/s** | **12. Research stakeholders considered in research design or conduct.** | **13. Strengths and limitations critically discussed** |
| --- | --- | --- | --- | --- | --- | --- | --- | --- | --- | --- | --- | --- | --- |
| **SRs and RERs** | | | | | | | | | | | | | |
| Jimenez Soto 2012 |  |  |  |  |  |  |  |  |  |  |  |  |  |
| Kaur 2019 |  |  |  |  |  |  |  |  |  |  |  |  |  |
| Zhao 2022 |  |  |  |  |  |  |  |  |  |  |  |  |  |
| Stratil 2020a |  |  |  |  |  |  |  |  |  |  |  |  |  |
| **Mixed methods studies** | | | | | | | | | | | | | |
| Otim 2014 |  |  |  |  |  |  |  |  |  |  |  |  |  |
| Leider 2014 |  |  |  |  |  |  |  |  |  |  |  |  |  |
| Baum 2011 |  |  |  |  |  |  |  |  |  |  |  |  |  |
| Hunter 2019 |  |  |  |  |  |  |  |  |  |  |  |  |  |
| Kapiriri 2022 |  |  |  |  |  |  |  |  |  |  |  |  |  |
| Bekemeier 2013 |  |  |  |  |  |  |  |  |  |  |  |  |  |
| Gilsdorf 2011 |  |  |  |  |  |  |  |  |  |  |  |  |  |
| Platonova 2010 |  |  |  |  |  |  |  |  |  |  |  |  |  |
| Marks 2013 |  |  |  |  |  |  |  |  |  |  |  |  |  |
| **Qualitative studies** | | | | | | | | | | | | | |
| Wanjau 2021 |  |  |  |  |  |  |  |  |  |  |  |  |  |
| Greaves 2017 |  |  |  |  |  |  |  |  |  |  |  |  |  |
| **Prioritisation exercises** | | | | | | | | | | | | | |
| Klamer 2021 |  |  |  |  |  |  |  |  |  |  |  |  |  |
| Balabanova 2011 |  |  |  |  |  |  |  |  |  |  |  |  |  |
| Graham 2016 |  |  |  |  |  |  |  |  |  |  |  |  |  |
| Frew 2020 |  |  |  |  |  |  |  |  |  |  |  |  |  |
| Mitton 2011 |  |  |  |  |  |  |  |  |  |  |  |  |  |
| Marsh 2012 |  |  |  |  |  |  |  |  |  |  |  |  |  |
| Suwantika 2021 |  |  |  |  |  |  |  |  |  |  |  |  |  |
| **Modelling/framework development and evaluation case studies** | | | | | | | | | | | | | |
| Lasry 2012 |  |  |  |  |  |  |  |  |  |  |  |  |  |
| Stratil 2020b |  |  |  |  |  |  |  |  |  |  |  |  |  |
| Hauck 2016 |  |  |  |  |  |  |  |  |  |  |  |  |  |
| Choi 2019 |  |  |  |  |  |  |  |  |  |  |  |  |  |
| Longfield 2012 |  |  |  |  |  |  |  |  |  |  |  |  |  |
| Simoes 2006 |  |  |  |  |  |  |  |  |  |  |  |  |  |
| Maciosek 2017 |  |  |  |  |  |  |  |  |  |  |  |  |  |
| Dahl 2015 |  |  |  |  |  |  |  |  |  |  |  |  |  |
